# Sequential enhancer state remodelling defines human germline competence and specification

**DOI:** 10.1038/s41556-022-00878-z

**Published:** 2022-04-11

**Authors:** Walfred W.C. Tang, Aracely Castillo-Venzor, Wolfram H. Gruhn, Toshihiro Kobayashi, Christopher A. Penfold, Michael D. Morgan, Dawei Sun, Naoko Irie, M. Azim Surani

**Affiliations:** 1 Wellcome Trust/Cancer Research UK Gurdon Institute, Henry Wellcome Building of Cancer and Developmental Biology, Cambridge, CB2 1QN, UK; 2 Physiology, Development and Neuroscience Department, University of Cambridge, Cambridge, CB2 3EL, UK; 3 Wellcome - MRC Cambridge Stem Cell Institute, Jeffrey Cheah Biomedical Centre, Puddicombe Way, Cambridge Biomedical Campus, Cambridge, CB2 0AW, UK; 4 Division of Mammalian Embryology, Center for Stem Cell Biology and Regenerative Medicine, The Institute of Medical Science, The University of Tokyo, Minato-ku, Tokyo 108-8639, Japan; 5 Center for Genetic Analysis of Behavior, National Institute for Physiological Sciences, Okazaki, Aichi 444-8787, Japan; 6 Centre for Trophoblast Research, University of Cambridge, Downing Site, Cambridge CB2 3EG, United Kingdom; 7 Cancer Research UK Cambridge Institute, University of Cambridge, Li Ka Shing Centre, Robinson Way, Cambridge, United Kingdom; 8 European Molecular Biology Laboratory, European Bioinformatics Institute, Wellcome Genome Campus, Cambridge, United Kingdom

## Abstract

Germline-soma segregation is a fundamental event during mammalian embryonic development. Here, we establish the epigenetic principles of human primordial germ cell (hPGC) development using in vivo hPGCs and stem cell models recapitulating gastrulation. We show that morphogen-induced remodelling of mesendoderm enhancers transiently confers the competence for hPGC fate, but further activation favours mesoderm and endoderm fates. Consistently, reducing the expression of the mesendodermal transcription factor (TF) OTX2 promotes the PGC fate. In hPGCs, SOX17 and TFAP2C initiate activation of enhancers to establish a core germline program, including the transcriptional repressor PRDM1 and pluripotency factors POU5F1 and NANOG. We demonstrate that *SOX17* enhancers are the critical components in the regulatory circuitry of germline competence. Furthermore, activation of upstream *cis*-regulatory elements by an optimised CRISPR activation system is sufficient for hPGC specification. We reveal an enhancer-linked germline TF network that provides the basis for the evolutionary divergence of mammalian germlines.

## Introduction

The fusion of sperm and egg during fertilisation initiates organismic development by generating a totipotent zygote, allowing transmission of genetic and epigenetic information to the offspring and over an evolutionary time scale^1^. Primordial germ cells (PGCs), the precursors of gametes, emerge in the embryo around the onset of gastrulation^2–4^, upon instructive signals inducing epigenetic and transcriptional responses for germline-soma segregation^5–10^. In mice, an integrated program temporally and spatially restricts germline competence, resulting in ~30 founder PGCs specified in response to bone morphogenetic proteins (BMP) signalling^11, 12^.

**Fig. 1:**
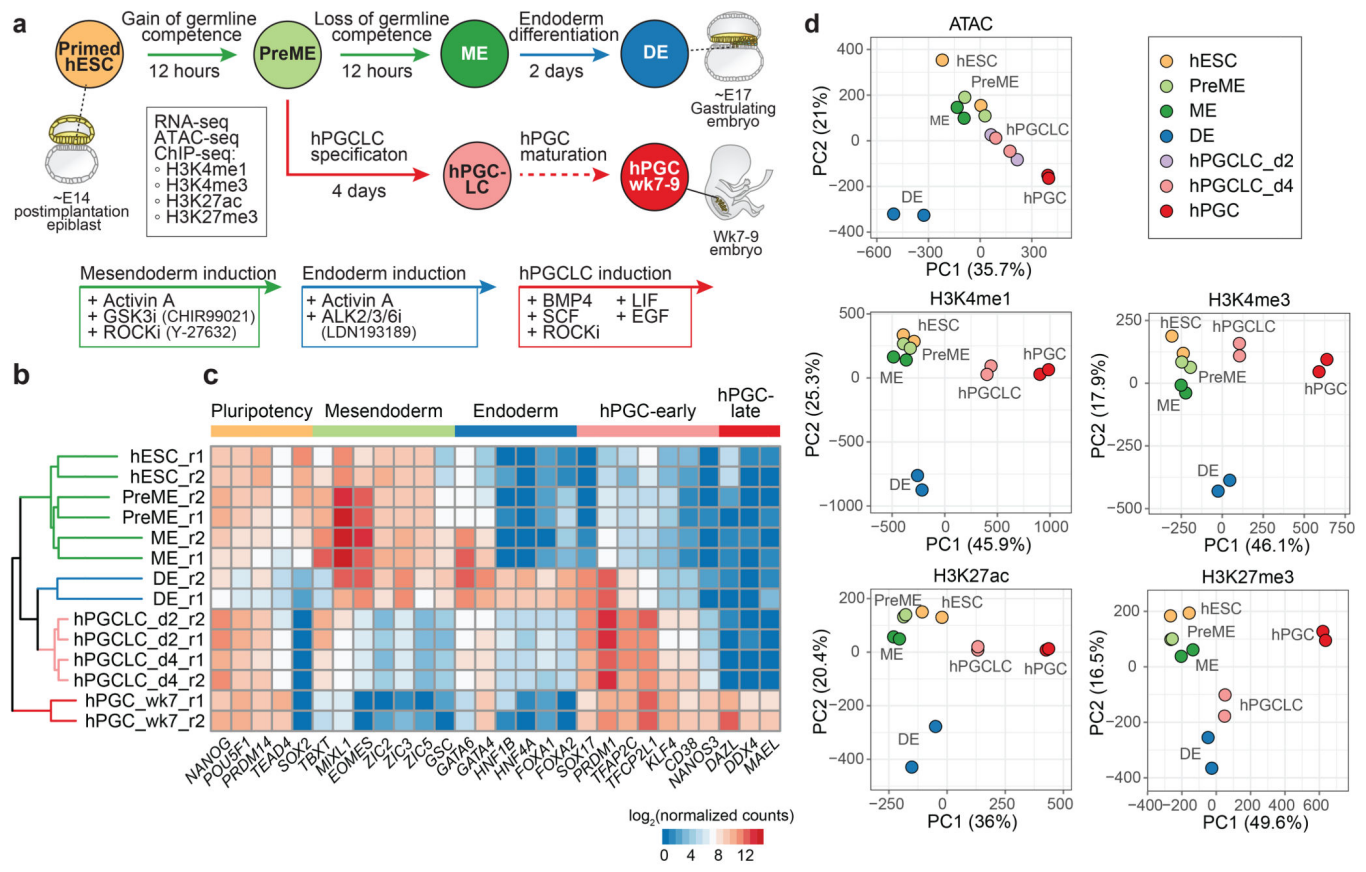
Genome-wide transcriptome and chromatin profiling revealed the trajectories of gastrulation and hPGC development. a, Generation and collection of in vitro and in vivo samples for RNA-seq, ATAC-seq, and histone modification ULI-NChIP-seq. E: embryonic day. b, Unsupervised hierarchical clustering of gene expression (RNA-seq) using all expressed genes. r: replicate, d: day, wk: week. c, Expression heat maps of lineage-specific genes. d, Principal component analysis of ATAC-seq, H3K4me1, H3K4me3, H3K27ac and H3K27me3 ChIP-seq signals (log2(normalized counts)) at combined peaks of all cell types (see Methods).

Since studies on nascent human PGCs (hPGCs) at week (wk) 2-3 of gestation are ethically and technically not feasible, we established *in vitro* models to investigate the molecular mechanisms driving hPGC specification^9^ (Fig. 1a). Briefly, human embryonic stem cells (hESCs), which equate to the post-implantation epiblast^13^, are differentiated towards mesendoderm (ME) fate by canonical WNT and ACTIVIN/NODAL signalling^9, 14, 15^. At ~12 hours (h), pre-mesendoderm (PreME) cells transiently gain competence for the germ cell fate and differentiate into primordial germ cell-like cells (hPGCLCs) in response to BMP4. PreME cells left to continue their progression form ME at 24h, lose germline competence and gain competence for definitive endoderm (DE) and mesoderm fates. This reductionist model allows us to dissect the molecular basis of transient competence for hPGCLC specification.

Using our *in vitro* models^9, 16^, we previously found a diverged TF network for hPGC fate from mouse^8, 17^, with SOX17, a known driver of DE formation, emerging as a critical regulator of hPGC fate^16, 18^. Consistently, SOX17-positive hPGCs were amongst the posterior primitive streak cells in a rare wk3 (Carnegie stage 7) gastrulating embryo^13^. Besides SOX17, PRDM1 (or BLIMP1) and TFAP2C are also essential for the hPGC fate^9, 16, 19–21^, with their expression and functions potentially continuing in migratory and gonadal germ cells *in vivo*^22–26^. Defining how these TFs promote hPGC specification and maturation is crucial for understanding germline development.

Here, we show how morphogens transiently confer human germline competence and direct cell fate choices by sequential epigenetic patterning of enhancer elements. Clustered regularly interspaced short palindromic repeats (CRISPR)-mediated modulation of enhancer activity reveals their importance for regulating critical TFs mediating germline development. Accordingly, an intricate enhancer-regulated TF network underpins hPGC specification and progression.

## Results

### Epigenetic trajectories upon germline-soma segregation

**Extended Data Fig. 1:**
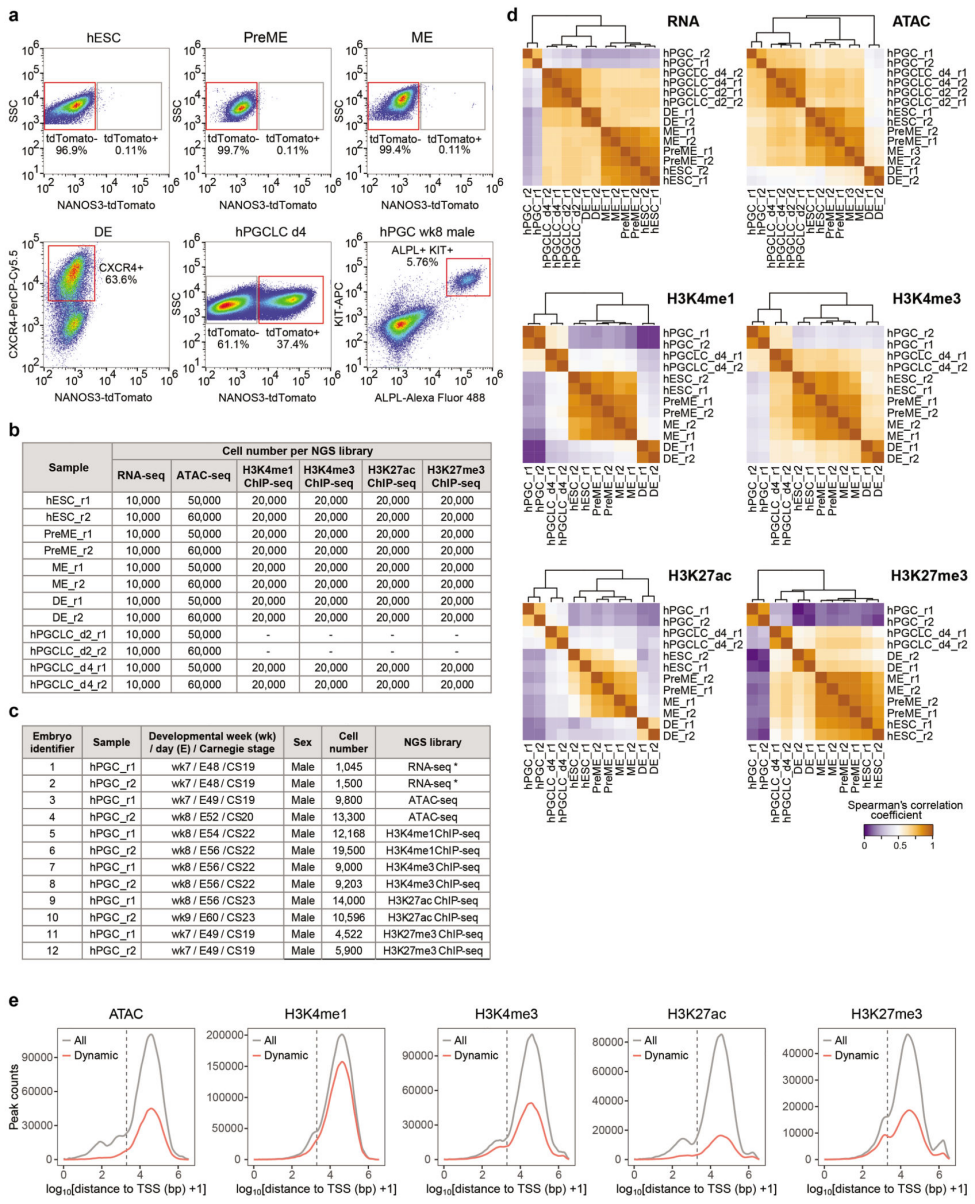
Sample collection and overview of transcriptomic and epigenomic data. a, Fluorescence-activated cell sorting (FACS) pseudocolor plots showing the cell populations collected for transcriptomic and epigenomic analysis (red gates). b, Table showing the hESC-derived cell types and the number of cells used to generate RNA-seq, ATAC-seq, and ChIP-seq libraries. c, Table showing details of the human embryos used for hPGC isolation and the number of hPGCs used to generate RNA-seq, ATAC-seq, and ChIP-seq libraries. Asterisks indicate RNA-seq samples published in a previous study^16^. d, Heatmaps showing Spearman’s correlation coefficient of gene expression, ATAC-seq, H3K4me1, H3K4me3, H3K27ac, and H3K27me3 ChIP-seq signals in biological replicates. For RNA expression, correlation was based on the log2(normalized counts) of the top 25% most variable protein coding genes and lincRNA. For ATAC-seq, signals (log2(normalized counts)) at combined peaks across the 6 cell types were used. For H3K4me1, H3K4me3, H3K27ac, and H3K27me3 ChIP-seq, signals (log2(normalized counts)) at 1 kb bins of combined peaks were used. The samples were clustered using (1 - Spearman’s correlation coefficient) as the distance measure (Ward’s method with optimal tree ordering). See Methods. e, Distance distribution between the summit of ATAC peaks or the centre of histone modification peaks and the closest TSS. Shown are all peaks and dynamic peaks with differential chromatin signals in the sample pairs shown in Extended Data Fig. 2d (log2(signal fold change) >1 and adjusted p-value <0.05). Note that most of the dynamic ATAC, H3K4me1, H3K4me3, H3K27ac and H3K27me3 peaks were >2 kb away (dotted line) from the TSS.

We investigate the epigenomic dynamics in our *in vitro* model, which simulates human gastrulation and germline formation^9^. Employing hESCs harbouring the highly specific germline reporter NANOS3-T2A-tdTomato, we examined cell state transitions towards PreME, ME, DE and hPGCLCs (Fig. 1a). We performed RNA sequencing (RNA-seq), transposase-accessible chromatin followed by sequencing (ATAC-seq)^27^, and ultra-low-input native chromatin immunoprecipitation followed by sequencing (ULI-NChIP-seq)^28^ for promoter- and enhancer-associated histone modifications (H3K4me1, H3K4me3, H3K27ac and H3K27me3) (Extended Data Fig. 1a,b, Supplementary Table 1). The hPGCLCs are at the nascent pre-migratory stage, which we compared with the closest available *in vivo* gonadal hPGCs from individual human male wk7-9 embryos (with ethical approval) (Extended Data Fig. 1c).

Unsupervised hierarchical clustering (UHC) of gene expression revealed three main branches; 1.) hESCs, PreME, and ME, 2.) DE, and hPGCLCs, and 3.) hPGCs formed a separate branch albeit clustering closest to hPGCLCs (Fig. 1b, Extended Data Fig. 1d). Robust expression of most pluripotency factors was observed in all cell types apart from DE, while *SOX2* expression diminished in ME and was absent in hPGCLCs and hPGCs ^9, 16, 22^. (Fig. 1c). There was substantial upregulation of mesendodermal genes, *TBXT* and *EOMES* in PreME, whereas *GSC, GATA4*, and *GATA6* were induced later in ME and co-expressed with endoderm TFs (e.g., *FOXA1*, *FOXA2* and *HNF4A*) in DE. Strong *SOX17* and *PRDM1* expression were detected in hPGCLCs, hPGCs, and DE. In hPGCLC and hPGC, there was the expression of *TFAP2C*, *NANOS3* and *CD38*, with naïve pluripotency factors *TFCP2L1* and *KLF4*^29, 30^, while *DAZL*, *DDX4* and *MAEL*, the meiosis-associated RNA binding proteins were expressed in the gonadal hPGCs^25, 26^.

Next, Spearman’s correlation and UHC of normalized signals at combined peak sets of all cell types showed ATAC, H3K4me1, H3K4me3, and H3K27ac signals exhibited a similar clustering pattern (Extended Data Fig. 1d). Accordingly, hESCs, PreME and ME formed one group separated from DE, whereas hPGCLCs and hPGCs clustered in another branch. Principal component analysis (PCA) of H3K4me1, H3K4me3, and H3K27ac signals linked germline trajectory to hESCs, hPGCLCs and hPGCs along principal component (PC) 1, and an endoderm trajectory connects hESCs, PreME, ME and DE along PC2 (Fig. 1d). However, PCA of H3K27me3 signals placed hPGCs away from hPGCLCs and other *in vitro* derived cells along PC1, whilst the endoderm trajectory along PC2 was preserved, reflecting potentially the global reduction of H3K27me3 and DNA demethylation^25^. Overall, the epigenomic trajectories were consistent with human gastrulation and germline establishment (Fig. 1a).

### Activation of enhancers underlies cell fate transitions

**Extended Data Fig. 2:**
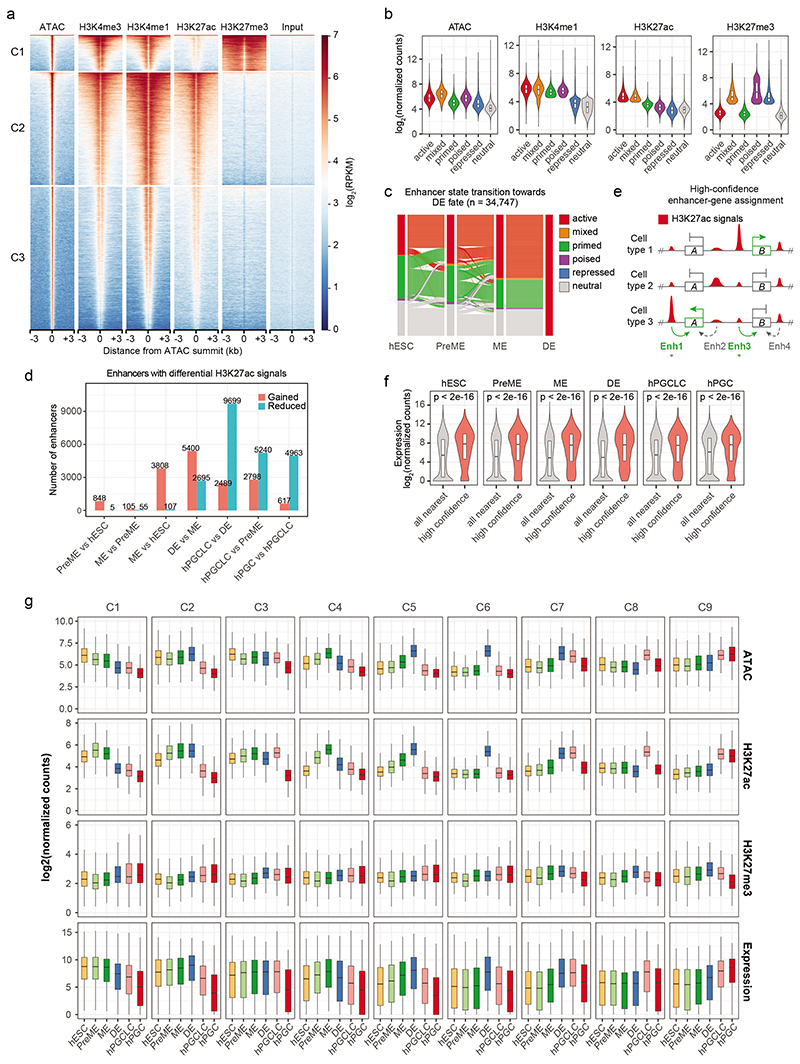
Characterisation of dynamically active enhancers. a, Chromatin profile heatmaps of ATAC, H3K4me3, H3K4me1, H3K27ac, H3K27me3 and input signals in hPGCLCs at ATAC-seq summit ± 3 kb. Segregation of ATAC-seq summits by K-means clustering using normalised chromatin mark signals. b, Distribution of chromatin mark signals at active, mixed, primed, poised, repressed, and neutral enhancers in hPGCLCs (see Fig. 2a). Enhancers per violin/box plot: 23255 active, 1288 mixed, 36999 primed, 5648 poised, 3984 repressed, 79290 neutral. Box plots depict the median, lower and upper hinges correspond to the 25th and 75th percentiles. c, Enhancer state transitions of DE-active enhancers. Distal OCRs not overlapping any histone modification peak in the analysed cell types were referred to as ‘neutral’ enhancers. d, Putative enhancers with differential H3K27ac levels (absolute(log2(fold change)) >1 and adjusted p-value < 0.05) in the indicated sample pairs. e, High confidence enhancer-gene associations. Putative enhancers were assigned to the nearest TSS. The relevance of the enhancer-gene pair was assessed by the Kendall’s rank correlation analysis between the enhancer H3K27ac signals and the expression levels of the associated genes across the 6 cell types and 2 replicates (see Methods). In the simplified model shown, the Enh1-geneA and Enh3-geneB pairs (green text and arrows) were identified as high confidence associations based on positive correlation between H3K27ac and gene expression levels. f, Expression levels of genes associated with active enhancers in different cell types. Compared to simply associating genes to the nearest active enhancer, high confidence active enhancer associated genes (Kendall rank correlation coefficient >0.3; empirical p-value < 0.05) exhibited significantly higher expression in all cell types. Two-sided Wilcoxon rank sum test with FDR correction. Gene number per violin/box plot (all nearest, high confidence): hESC (8686, 1279), PreME (9782, 1413), ME (11494, 1853), DE (12279, 2224), hPGCLC (9954, 1726), hPGC (6612, 1024). Box plot organisation as in Extended Data Fig. 2b. g, Distribution of ATAC, H3K27ac and H3K27me3 signals in dynamically active enhancers and high confidence target gene expression. Enhancers were segregated into nine clusters (Fig. 2c). Enhancer per clusters as in Fig. 2c. Box plot organisation as in Extended Data Fig. 2b.

**Fig. 2:**
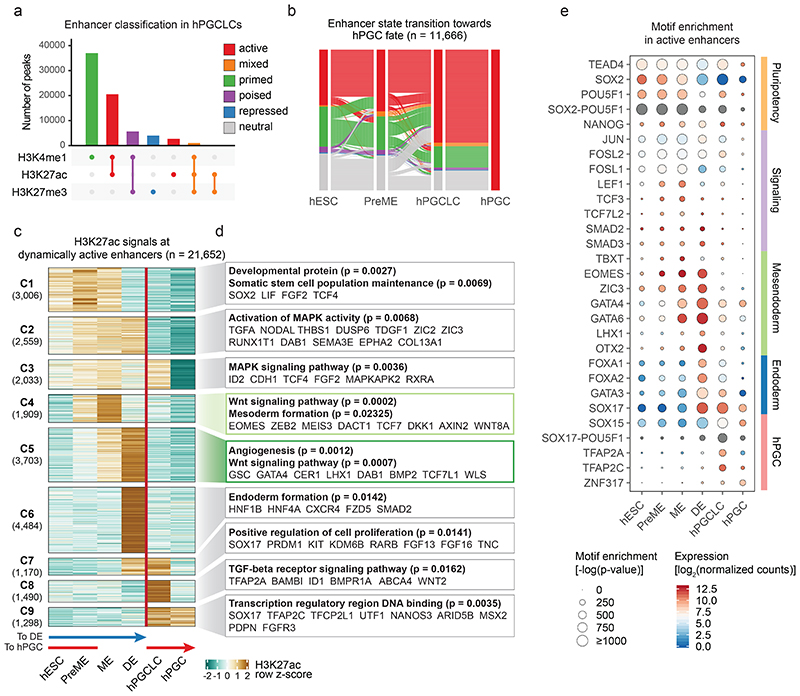
Dynamic activation of enhancers underlies cell fate transitions. a, Classification of enhancers in hPGCLCs by the intersection of histone modification peaks at combined distal open chromatin regions (OCRs) (ATAC summit ± 500 bp). Note that ‘neutral’ enhancers (distal OCRs that did not overlap with any histone modification peak in the cell type of interest) were not shown. b, Alluvial plots showing enhancer state transitions of hPGC-active enhancers. Colour key is shown in a. c, K-means clustering of dynamically active enhancers into 9 clusters by H3K27ac signals. Dynamically active enhancers were defined as enhancers that were active in any cell type with differential H3K27ac signals between the contrasting pairs shown in Extended Data Fig. 2d. d, Gene ontology enrichment analysis (DAVID 6.8)^81^ on the high confidence target genes in each dynamically active enhancer cluster. The representative terms and representative genes are shown. The full enrichment list is provided in Supplementary Table 2. e, Dotplots showing the enrichment of representative TF motifs in active enhancers of each cell type. Dot size represents motif enrichment significance (-log(p-value)). Dot colour indicates expression levels of the corresponding TFs.

Most regions with differential epigenetic signals were 10-100 kb away from the nearest transcription start site (TSS) (Extended Data Fig. 1e), featuring open chromatin (ATAC), H3K4me1 and H3K27ac modifications, the hallmarks of enhancers^31^ (Extended Data Fig. 2a). To identify enhancer dynamics for the establishment of somatic and germ cell fates, we combined 150,464 distal nucleosome-free open chromatin regions (OCRs) (>1 kb away from TSS), which harbour TF and chromatin remodeler binding sites^32^. Enhancers were classified as active, mixed, primed, poised, repressed, and neutral based on general enhancer mark H3K4me1, P300-CBP-associated active H3K27ac and Polycomb Repressive Complex 2 (PRC2)-associated H3K27me3 occupancy (Methods, Fig. 2a, Extended Data Fig. 2b)^33–36^.

Tracing the activation of enhancers towards hPGC and DE fates, we found around 40% of enhancers ‘active’ in hPGCs (hPGC-active enhancers) were already active in hESCs, PreME and hPGCLCs (Fig. 2b), while the remaining hPGC-active enhancers were primed (around 1/3) or neutral (around 1/4) in hESCs and became progressively activated in PreME, hPGCLCs or hPGCs. However, most DE-active enhancers were activated during the ME to DE transition (Extended Data Fig. 2c,d), suggesting a drastic change in the chromatin landscape.

K-means clustering of dynamically active enhancers exhibiting differential H3K27ac occupancy revealed 21,652 enhancers falling into nine groups (Fig. 2c). Cluster (C) 1 enhancers had strong H3K27ac signals in hESCs, PreME and ME, but not in DE and germ cells. Gene ontology enrichment analysis of high-confidence target genes (Methods, Extended Data Fig. 2e,f) suggested that C1 enhancers regulated genes encoding ‘developmental proteins’ and ‘somatic stem cell population maintenance’, including *SOX2*, *FGF2* and *LIF* (Fig. 2d and Supplementary Table 2). These hESC-associated genes remained highly expressed during mesendoderm formation but were downregulated in DE, hPGCLCs and hPGCs (Extended Data Fig. 2g). C6 enhancers were activated specifically in DE and associated with genes involved in ‘endoderm formation’ (e.g. *HNF1B* and *CXCR4*) (Fig. 2c,d, Extended Data Fig. 2g). Notably, C9 enhancers were associated with germ cell genes (e.g., *SOX17*, *TFAP2C*, *UTF1*, *NANOS3*, and *PDPN*), showing strong H3K27ac enrichment in hPGCLCs and hPGCs. Motif enrichment analysis on hPGCLC-active enhancers suggested that SOX17, TFAP2C and POU5F1 might activate and maintain germline enhancers (Fig. 2e).

**Extended Data Fig. 3:**
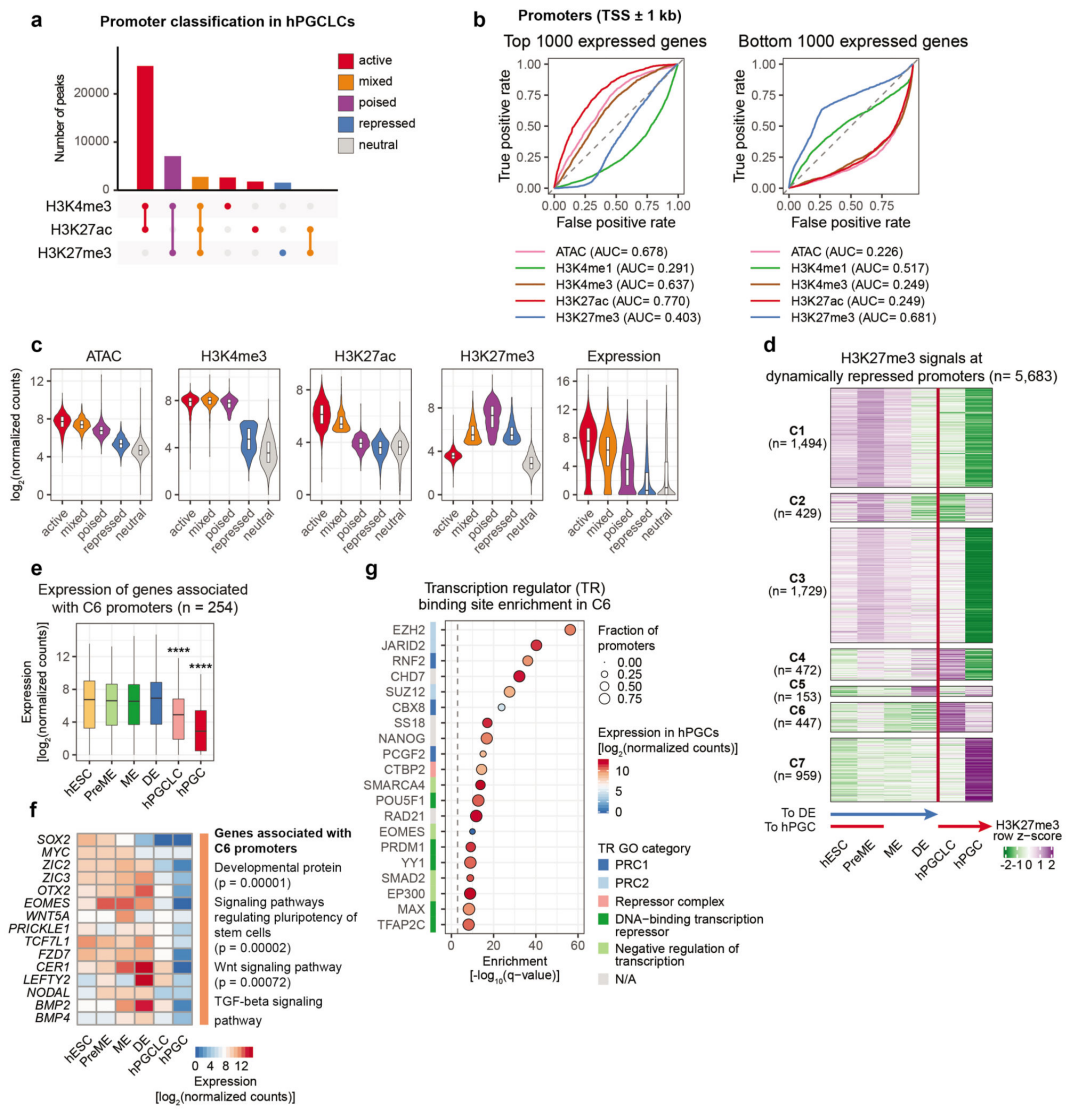
Characterisation of dynamically active and repressed promoters during hPGC development. a, Promoter classification in hPGCLCs based on the intersection of histone modification peaks at promoter regions (TSS ± 1 kb). b, Receiver operating characteristic (ROC) curves of ATAC, H3K4me3, H3K4me1, H3K27ac, and H3K27me3 signals at promoter (TSS ± 1 kb) as predictors of gene activity in hPGCLCs. The top 1000 (top panel) or the bottom 1000 (bottom panel) expressed genes were used as positives. Promoters not overlapping any chromatin peak were excluded. Note that H3K27ac (area under the curved (AUC) of top genes = 0.770) and H3K27me3 (AUC of bottom genes = 0.681) were the best predictors of expressed and repressed genes, respectively. c, Distribution of chromatin mark signals at active, mixed, poised, repressed, and neutral promoters and the expression of their associated genes in hPGCLCs. Promoter number: 30261 active, 2833 mixed, 7089 poised, 1579 repressed, 19832 neutral. Associated gene number: 12629 active, 1526 mixed, 3662 poised, 1144 repressed, 13038 neutral. Box plot organisation as in Extended Data Fig. 2b d, K-means clustering of dynamically repressed promoters into 7 clusters (C) by H3K27me3 signals. Dynamically repressed promoters were promoters that exhibited ‘mixed’, ‘poised’ or ‘repressed’ state (see Methods) in any cell type with differential H3K27me3 signals. e, Box plots showing expression levels of genes associated with the C6 dynamically repressed promoters in c. **** p-value < 0.00001 (Wilcoxon rank sum test adjusted by the Holm method) in marked against each unmarked cell type. Box plot organisation as in Extended Data Fig. 2b f, Heatmaps showing the expression levels of representative genes associated with C6 in c. The right panel shows the representative enriched gene ontology terms. g, Dot plots showing the enrichment of transcription regulator (TR) binding site in the dynamic repressed promoters in C6 in c. The top 20 enriched TRs (out of 1,135 in the ReMap2020 database^42^) are shown. TRs were annotated against 5 gene ontology terms associated with repressive functions. The dot size represents promoter fraction overlapping with the TR binding sites. The dot colour indicates the expression levels of the enriched transcription regulators in hPGCs.

Next, we defined promoters (TSS ± 1 kb) as active, mixed, poised, repressed and neutral based on their H3K4me3, H3K27ac and H3K27me3 occupancy (Methods, Extended Data Fig. 3a-c). Notably, promoters gaining H3K27me3 during the PreME-hPGCLC transition showed reduced expression in hPGCLCs and enrichment for the PRDM1 motif, which might indicate direct PRDM1-mediate promoter repression (Extended Data Fig. 3 d-g).

### SOX17 and PRDM1 drive hPGC fate interdependently

**Extended Data Fig. 4:**
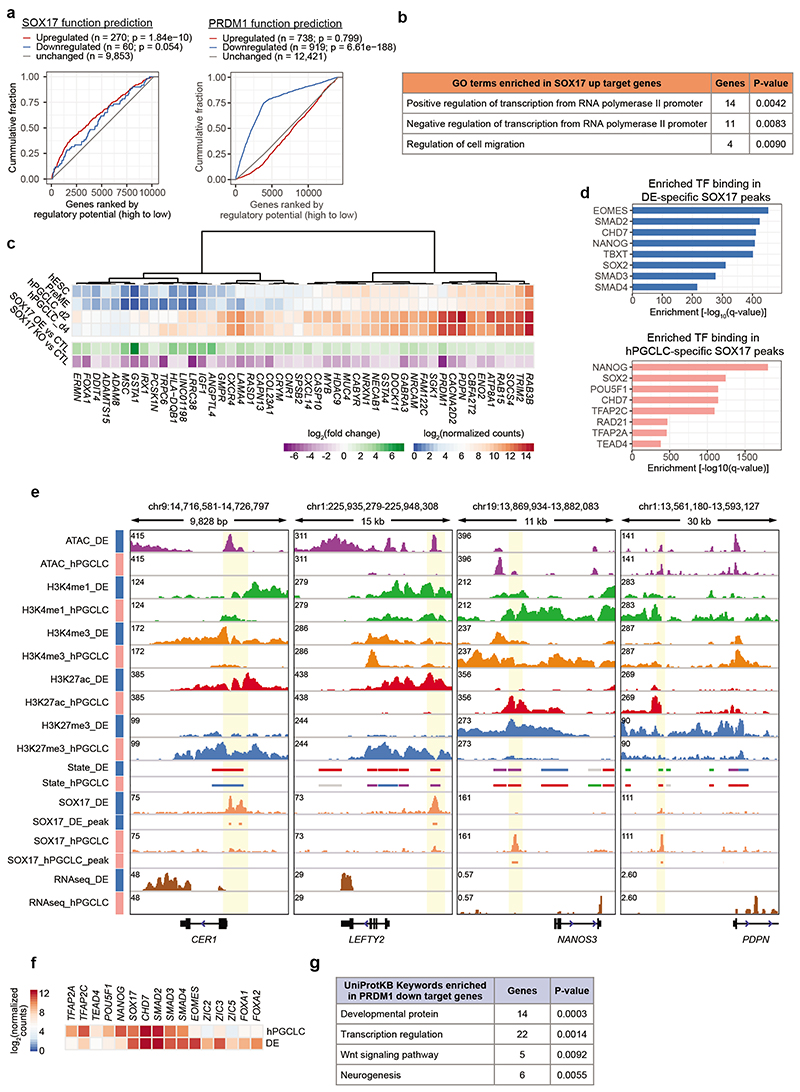
Direct targets of SOX17 in hPGCLCs and DE. a, Cumulative distribution function plot showing the functional prediction of SOX17 and PRDM1. The ChIP peaks of SOX17/PRDM1 in hPGCLCs were assigned to genes with TSS within 100 kb of the peak summits. A regulatory potential score was calculated for each gene based on the distance between the peak summit and the TSS^96^. The genes were then divided into upregulated, downregulated and unchanged according to their expression patterns upon SOX17 or PRDM1 overexpression. Cumulative distribution function plot was generated for each group with genes ranked by decreasing regulatory potential. A one-tailed Kolmogorov-Smirnov test was used to determine the statistical significance between the differentially expressed groups and the unchanged group. Note that upregulated genes (but not downregulated genes) upon SOX17 induction have a significantly higher tendency to be bound by SOX17. In contrast, genes downregulated upon PRDM1 overexpression tend to be bound by PRDM1. b, GO biological process terms that were enriched in SOX17 direct up targets (red dots in Fig. 3d) c, Expression heatmap of SOX17 direct up target genes. Shown are the genes which were (1) upregulated both by SOX17 alone (log2(fold change) >1 and adjusted p-value <0.05 between Dex-treated and non-treated 12h PreME aggregates); (2) upregulated by cytokines (log2(fold change) >2 and adjusted p-value <0.05 between day 2 hPGCLCs and PreME); and (3) downregulated in *SOX17* KO (log2(fold change) >1 and adjusted p-value <0.05 between *SOX17* KO day 2 aggregate and WT day 2 aggregate)^20^. d, Top eight TFs with binding sites (ReMap2020) enriched in hPGCLC-specific and DE-specific SOX17 peaks. e, Genome browser snapshots showing the epigenetic landscape of DE-specific (*CER1* and *LEFTY2*) and hPGCLC-specific (*NANOS3* and *PDPN*) SOX17-bound gene targets. f, Heatmap showing expression of genes associated with the top enriched motifs (Fig. 3h) and the top enriched TF binding sites (in d) in hPGCLC-specific and DE-specific SOX17 peaks. g, UniProtKB Keywords that were enriched in PRDM1 direct down targets (blue dots in Fig. 3i).

**Fig. 3:**
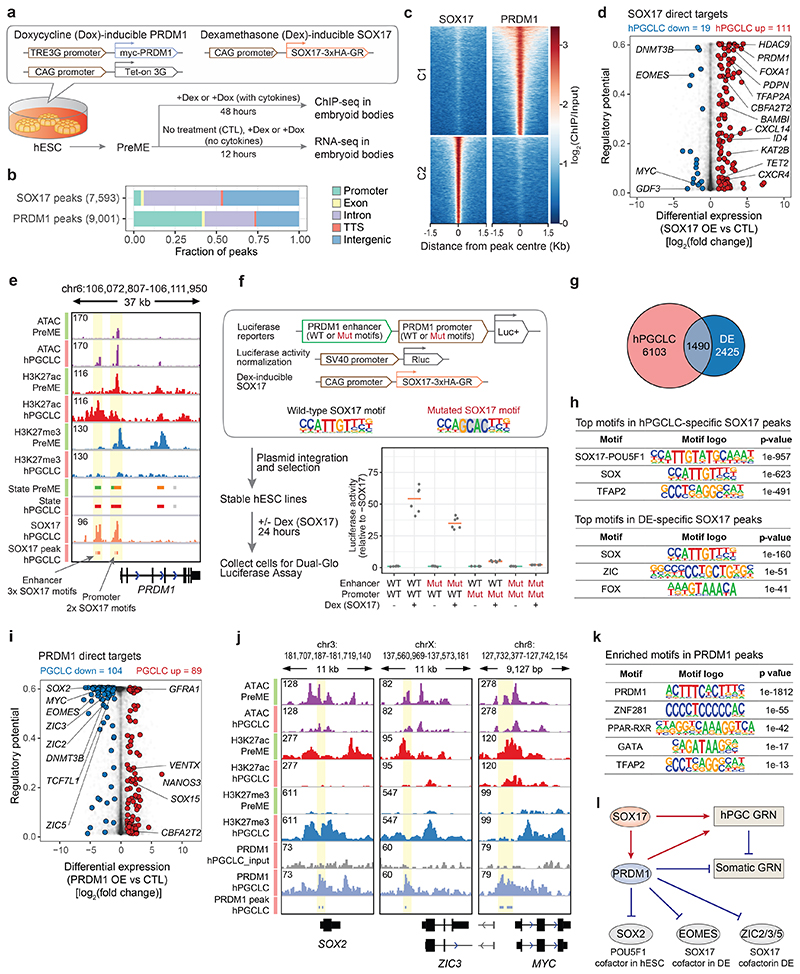
SOX17 and PRDM1 drive hPGC fate interdependently. a, Experimental design to identify direct targets of SOX17 and PRDM1. b, Genomic distribution of the SOX17 and PRDM1 peaks. TTS: Transcription termination site. c, K-means clustering of SOX17 and PRDM1 ChIP-seq signals in hPGCLCs. d, Direct targets of SOX17 in hPGCLCs. The regulatory potential of each gene (the higher the score, the closer is the distance between peak summit and TSS) was plotted against its expression pattern in PreME aggregates after SOX17 overexpression. Red dots: genes that are upregulated by SOX17 alone (Dex-treated vs. non-treated) and by cytokines (day 2 hPGCLCs vs. PreME). Blue dots: genes that are downregulated by SOX17 alone and by cytokines. e, Binding of SOX17 to the *PRDM1* enhancer and promoter. f, Direct regulation of the *PRDM1 cis*-regulatory elements by SOX17. The *PRDM1* enhancer and/or the promoter were cloned into a vector containing a firefly luciferase reporter. The core ‘ATTGT’ SOX motifs were mutated into ‘AGCAC’. Each reporter plasmid was stably transfected into hESCs, together with a Dex-inducible SOX17-cGR plasmid^9^. Luciferase assays were performed in hESCs 24h after ± Dex treatment. Representative result with technical replicates shown as data points and median depicted as horizontal bar; n=5 (- Dex) n=6 (+ Dex). Experiment was repeated independently for 3 times with similar results. Rluc: Renilla luciferase. g, The intersection of SOX17 peaks in hPGCLCs and DE. h, Top motifs enriched in hPGCLC-specific and DE-specific peaks by HOMER (cumulative binomial distributions) i, Direct targets of PRDM1 in hPGCLCs. The regulatory potential of each gene was plotted against its expression pattern in PreME aggregates after PRDM1 overexpression. Red dots: genes that are upregulated by PRDM1 alone (Dox-treated vs. non-treated) and by cytokines (day 2 hPGCLCs vs. PreME). Blue dots: genes that are downregulated by PRDM1 alone and by cytokines. j, Binding of PRDM1 to their direct targets. k, The representative motifs enriched in PRDM1 peaks in hPGCLCs. l, The interdependent relationship of SOX17 and PRDM1 in hPGCLC specification. GRN: gene regulatory network.

To investigate SOX17 and PRDM1 function during hPGCLC specification, we employed a transgenic hESC line allowing doxycycline (Dox)-inducible Myc-tagged *PRDM1* and dexamethasone (Dex)-inducible haemagglutinin (HA)-tagged *SOX17* expression to conduct ChIP-seq in hPGCLCs^9^ (Fig. 3a). Notably, SOX17 and PRDM1 peaks showed minimal overlap, with SOX17 been predominantly found at distal intergenic and intronic regions (>90%), while PRDM1 exhibited pronounced promoter binding (Fig. 3b,c). To identify the direct transcriptional response triggered by SOX17 or PRDM1, we treated PreME aggregates with Dox or Dex (without cytokines) for 12 h and performed RNA-seq (Fig. 3a). Integrated analysis of ChIP-seq peaks and differential gene expression revealed SOX17 functioned mainly as transcriptional activator, whereas PRDM1 served primarily as transcriptional repressor during hPGCLC induction (Extended Data Fig. 4a). SOX17 directly upregulated well-known PGC genes, including *PRDM1*, *CBFA2T2*^37, 38^, Tet methylcytosine dioxygenase *TET2*, *PDPN* and *CXCR4*^39^ (Fig. 3d, Extended Data Fig. 4b,c, Supplementary Table 3).

Notably, SOX17 was bound to the *PRDM1* promoter and a ~6.2 kb upstream putative enhancer; both containing multiple SOX-binding motifs (Fig. 3e). Luciferase reporter assays in hESCs harbouring an inducible *SOX17* transgene showed that SOX17 strongly activated the *PRDM1* enhancers and promoters, which was abrogated by mutations in their SOX motifs, indicating that SOX17 directly upregulates *PRDM1* (Fig. 3f).

Importantly, SOX17 is critical for establishing both hPGC and DE fates^16, 40, 41^, where we found largely different SOX17 binding profiles (Fig. 3g, Extended Data Fig. 4g). Motif enrichment and transcriptional regulator binding site enrichment analyses^42^ of the SOX17 peaks suggested putative cell-type specific SOX17 cofactors including POU5F1, NANOG and TFAP2C in hPGCLCs, and EOMES, SMAD2/3/4, FOXA1/A2 and ZIC2/3/5 in DE (Fig. 3h, Extended Data Fig. 4d,e). In hPGCLCs, PRDM1 directly bound promoters of genes involved in the development, WNT signalling and neurogenesis, and confers repression of these genes in PreME aggregates upon PRDM1 overexpression (Fig. 3i, Extended Data Fig. 4f, Supplementary Table 4). *EOMES* and *ZIC2/3/5*, the putative SOX17 cofactors in DE, were amongst the direct targets repressed by PRDM1, along with *SOX2* (Fig. 3i,j), a cofactor of POU5F1 in regulating pluripotency genes (Fig. 2e)^43^. *SOX2* repression by PRDM1, and potentially by BMP and WNT signalling^44^, likely allows POU5F1 to partner with SOX17 resulting in redistribution from SOX2-POU5F1 canonical to compressed SOX17-POU5F1 motifs to activate hPGC genes^45^. PRDM1 potentially mediates gene repression through cofactors, e.g., GATA or TFAP2 TFs (Fig. 3k). In sum, SOX17 directly activates *PRDM1*, which represses pluripotency- and DE-associated TFs to facilitate SOX17’s function in hPGC specification (Fig. 3l); SOX17 and PRDM1 promote the hPGC transcriptional program interdependently without cooperative binding.

### Roles of TFAP2C, SOX17 and PRDM1 in hPGCLCs

**Extended Data Fig. 5:**
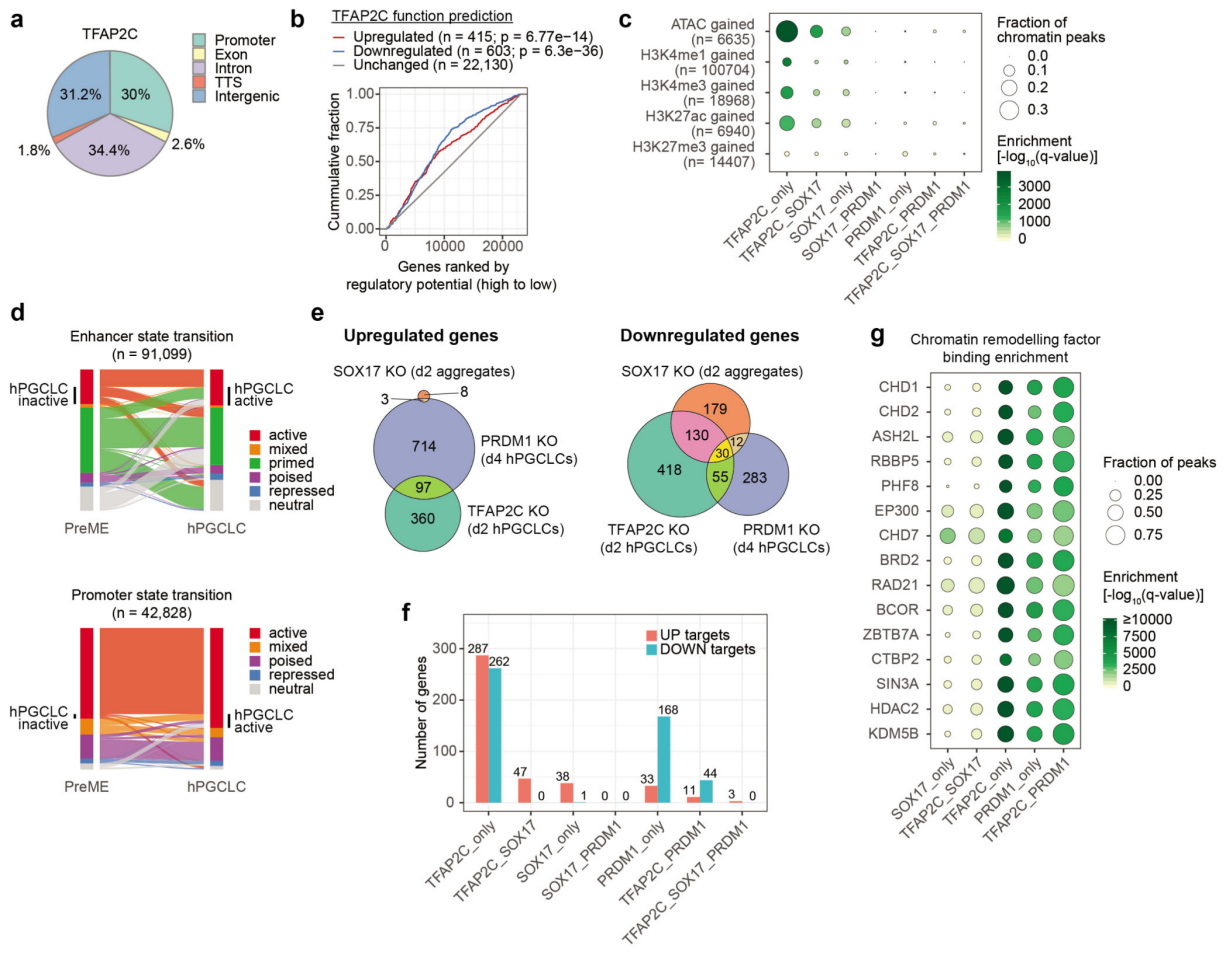
Direct target genes of TFAP2C, SOX17 and PRDM1. a, Genomic distribution of the TFAP2C peaks in hPGCLC aggregates^46^. b, Cumulative distribution function plot showing the functional prediction of TFAP2C. The TFAP2C peaks were assigned to genes with TSS within 100 kb of the peak summits. A regulatory potential score was calculated for each gene based on the distance between the peak summit and the TSS^96^. The genes were then divided into three groups (upregulated, downregulated and unchanged) according to their expression patterns in *TFAP2C* KO day2 hPGCLCs versus the wild-type control^20^. Cumulative distribution function plot was generated for each group with genes ranked by decreasing regulatory potential. A one-tailed Kolmogorov-Smirnov test was used to determine the statistical significance between the differentially expressed groups and the unchanged group. c, The enrichment of TFAP2C, SOX17 and PRDM1 peaks in genomic loci that gained ATAC, H3K4me1, H3K4me3, H3K27ac or H3K27me3 signals during the PreME to hPGCLC transition. The TF peaks were categorized into seven cooperativity classes as in Fig. 4a. The dot size represents the fraction of chromatin peaks that overlapped with the TF peaks. Dot color indicates enrichment significance. d, Alluvial plots showing the enhancer (upper panel) and promoter (lower panel) state transition from PreME to hPGCLC. The enhancers/promoters that became active/inactive in hPGCLCs were used for TF binding enrichment analysis in Fig. 4b. e, Venn diagram showing the intersection of upregulated and downregulated genes in *SOX17*, *TFAP2C* and *PRDM1* KO hPGCLCs/aggregate^20^. Upregulated and downregulated genes were defined as (log2(fold change versus wild-type control) >1 and adjusted p-value < 0.05) and (log2(fold change versus wild-type control) <(-1) and adjusted p-value < 0.05), respectively. f, The number of direct up and down target genes of TFAP2C, SOX17 and PRDM1 based on their cooperative binding. g, Enrichment of chromatin remodelling factor binding sites in TFAP2C, SOX17 and PRDM1 peaks in hPGCLCs. The y-axis shows the chromatin remodelling factors that were amongst the top 10 enriched transcriptional regulators (ReMap2020) in any of the five peak sets (x-axis).

The Transcription Factor AP-2 (TFAP2) DNA-binding motif was overrepresented within the SOX17 and PRDM1 peaks in hPGCLCs (Fig. 3h,k). Of the five TFAP2 family members, upregulation of TFAP2C is essential for specifying hPGCLC^9, 16, 19, 20^. Analysis of TFAP2C ChIP-seq data of day four hPGCLC aggregates^46^ revealed ~30,000 TFAP2C peaks evenly distributed between promoters, intronic and intergenic regions (Extended Data Fig. 5a). Integrated analysis of WT and TFAP2C knockout (KO) hPGCLCs^20^ revealed that TFAP2C acted both as a transcriptional activator and a repressor (Extended Data Fig. 5b).

**Fig. 4:**
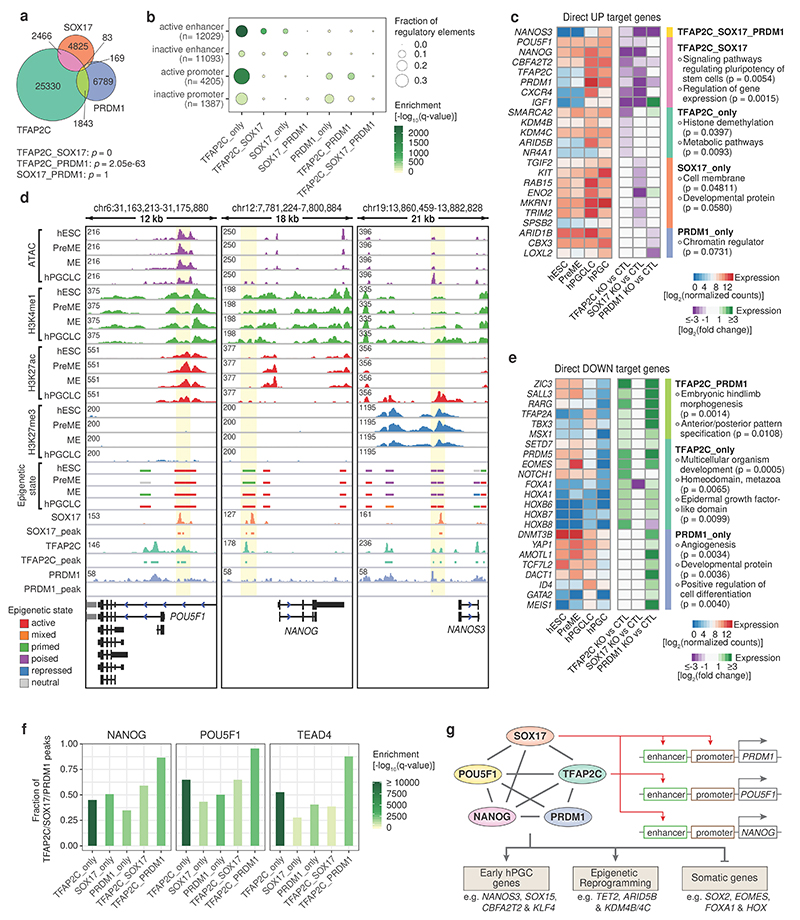
Combinatorial and individual roles of TFAP2C, SOX17 and PRDM1 in epigenetic regulation of target genes in hPGCLCs. a, The intersection of TFAP2C, SOX17 and PRDM1 peaks in hPGCLC aggregates. Statistical significance of overlap was determined by hypergeometric test. b, The enrichment of TFAP2C, SOX17 and PRDM1 peaks in promoters and enhancers that became active or inactive during the PreME to hPGCLC transition (see Extended Data Fig. 5d). The TF peaks were categorized into seven cooperativity classes as in a. Dot size represents the fraction of enhancers/promoters that overlapped with the TF peaks. c, The direct up target genes of TFAP2C, SOX17 and PRDM1. The heatmaps show the expression of representative target genes during hPGC development (left) and the expression pattern in *TFAP2C* (day 2), *SOX17* (day 2) and *PRDM1* (day 4) knockout (KO) hPGCLCs/aggregates versus wild-type control (CTL) (middle). The representative gene ontology terms enriched in the direct target genes based on the binding cooperativity of TFAP2C, SOX17 and PRDM1 are shown on the right. d, Genome browser snapshots of representative TFAP2C, SOX17 and PRDM1 direct up target genes. e, The direct down target genes of TFAP2C, SOX17 and PRDM1 and the representative gene ontology terms. f, Enrichment of NANOG, POU5F1, TEAD4 binding sites (ReMap2020 non-redundant peaks) in TFAP2C, SOX17 and PRDM1 peaks in hPGCLCs. g, The enhancer-linked TF network that establishes the hPGC program.

We observed substantial overlap between TFAP2C and SOX17 (2,466) and between TFAP2C and PRDM1 peaks (1,843), but little co-binding amongst the three factors (83) (Fig. 4a). Strikingly, TFAP2C alone bound to 39% of the loci gaining accessibility during hPGCLC induction, while TFAP2C-SOX17 together and SOX17 alone accounted for 13% and 6%, respectively (Extended Data Fig. 5c). Cross-referencing with our chromatin state maps, the bound sites of TFAP2C alone (21%), SOX17 alone (4%) and TFAP2C-SOX17 (6%) together overlapped more than 30% of enhancers activated during the PreME to hPGCLC transition (Fig. 4b, Extended Data Fig. 5d). Besides being a pioneering TF^47, 48^, TFAP2C might also contribute to promoter activation and promoter repression, both alone and with PRDM1 (Fig. 4b).

To identify individual and cooperative direct target genes of SOX17, TFAP2C and PRDM1, we integrated the DNA profiles of the three TFs with enhancer and promoter epigenetic state maps and loss-of-function RNA-seq data^20^ (Extended Data Fig. 5e,f, Supplementary Table 5,6, Methods). Among the only three cooperative targets of SOX17, TFAP2C, and PRDM1 was *NANOS3*, a conserved metazoan germ cell gene (Fig. 4c,d). TFAP2C-SOX17 manifestly cooperated to directly upregulate/sustain the expression of core pluripotency factors *POU5F1* and *NANOG* and the transcriptional repressors, *PRDM1* and *CBFA2T2*. Interestingly, TFAP2C promoted upregulation of H3K9 demethylases *KDM4B*, *KDM4C* and *ARID5B*, which might trigger H3K9me2 erasure and chromatin reorganization in hPGCs^25, 49^. TFAP2C and PRDM1 directly mediated the expression of the core components of chromatin remodelling BAF (SWI/SNF) complex *SMARCA2* and *ARID1B*, respectively, which maintains lineage-specific enhancers^50^. Furthermore, PRDM1 alone or with TFAP2C repressed somatic genes involved in embryonic development, anterior/posterior patterning, and cell differentiation (Fig. 4e, Supplementary Table 6). TFAP2C alone repressed homeodomain genes (e.g., *HOXA1*, *HOXB6* and *HOXB7*) and epidermal growth factor-like domain genes (e.g., *NOTCH1* and *LAMA1*).

Next, we intersected the cooperative peak sets with DNA binding profiles of 1,135 transcription regulators in the ReMap2020 database^42^. Strikingly, 28-88% of SOX17/TFAP2C/PRDM1 individual and combinatorial peaks overlapped with the binding sites of the pluripotency factors POU5F1 and NANOG, and of the trophectoderm factor TEAD4 (Fig. 4f), which showed robust expression in both hESCs and hPGCs (Fig. 1b). In hESCs, TEAD4, a key effector of Hippo signalling pathway^51, 52^, partners with POU5F1 to repress mesendoderm enhancers^53^. However, their functions and crosstalk with SOX17, TFAP2C and PRDM1 in hPGCs remain to be elucidated.

In summary, SOX17 and TFAP2C initially activated or sustained the expression of crucial TFs, including PRDM1, POU5F1 and NANOG; these, in turn, cooperated with SOX17, TFAP2C and epigenetic remodelers (Extended Data Fig. 5g) to shape the chromatin landscape towards hPGC fate (Fig. 4g).

### Enhancer-promoter cooperation regulates core hPGC TFs

**Extended Data Fig. 6:**
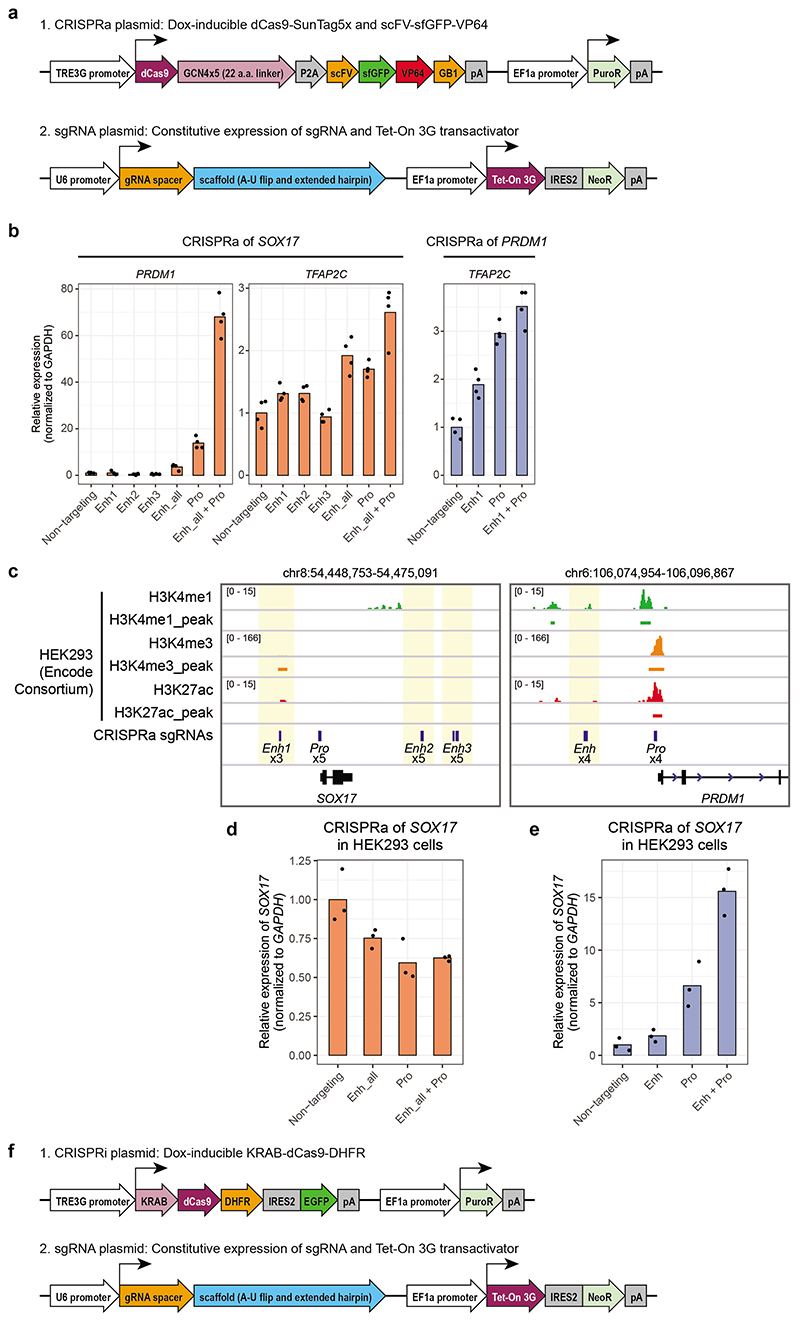
Inducible CRISPR activation and interference systems for activation and repression of hPGC TFs. a, The piggyBAC plasmids encoding an optimized doxycycline-inducible dCas9-SunTag-VP64 CRISPR activation system. Upon integration of both the CRISPRa plasmid and the sgRNA plasmid into the genome and Dox treatment, the Tet-On 3G doxycycline-binding transactivator protein encoded in the sgRNA plasmid will drive the transcription of dCas9-GCN4x5-P2A-scFV-sfGFP-VP64 through the TRE3G promoter. After translation of the mRNA, the recombinant protein will be split into dCas9-GCN4x5 and scFV-sfGFP-VP64 through the P2A self-cleaving peptide. Subsequently, the dCas9-GCN4x5 will be guided to enhancer/promoter by the constitutively expressed sgRNA and recruit up to 5 copies of the scFV-sfGFP-VP64 recombinant transactivator. To improve epigenome editing efficiency, the GCN4 epitopes were separated by optimized 22-amino-acid linkers^55^. To increase sgRNA expression and to enhance sgRNA-dCas9 affinity, a sgRNA scaffold with an A-U flip and extended hairpin was used^56^. b, RT-qPCR showing CRISPR activation of *SOX17* (2 days Dox treatment in hESCs) induced *PRDM1* and *TFAP2C* mRNA expression. Activation of *PRDM1* also upregulated *TFAP2C*. Average of 4 biological replicates, with individual replicates shown as data points. c, The epigenetic landscape of the *SOX17* and *PRDM1* loci in hESC, PreME, hPGCLCs and HEK293 cells. Note that the *SOX17* locus in HEK293 cells does not bear H3K4me1, H3K4me3 and H3K27ac marks. For CRISPR activation (CRISPRa) assay, 3-5 sgRNAs were used to activate each putative enhancer (highlighted) and promoter. d-e, RT-qPCR of *SOX17* and *PRDM1* following CRISPR activation of enhancers and promoters in HEK293 cells. HEK293 cells were transiently transfected with CRISPRa (dCas9-Suntag-VP64) and sgRNA plasmids and treated with Dox for 2 days. GFP-positive cells (expressing dCas9-Suntag and scFV-sfgFP-VP64) were isolated for RT-qPCR. Average of 3 technical replicates, with individual replicates shown as data points. Assay has been performed two times independently with similar results. f, The piggyBAC plasmids encoding a re-engineered doxycycline-inducible KRAB-dCas9-DHFR CRISPR interference system (also see Fig. 6a).

**Fig. 5:**
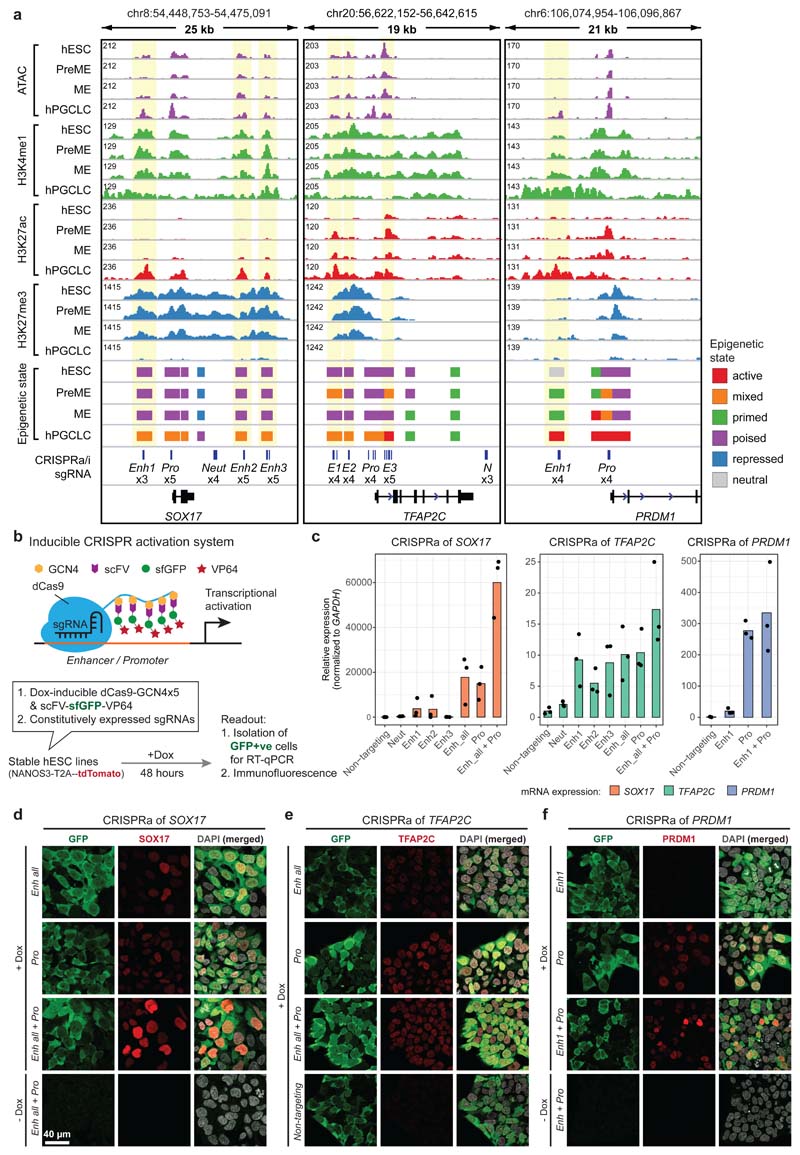
Enhancer and promoter trigger expression of core hPGC TFs synergistically. a, The epigenetic landscape of the *SOX17*, *TFAP2C* and *PRDM1* loci in PreME and hPGCLCs. For CRISPR activation (CRISPRa) assay, 3-5 sgRNAs were used to activate or repress each putative enhancer (Enh or E) (highlighted) and promoter (Pro or P). “Neutral” regions (Neut or N) which do not bear enhancer signature were chose as negative controls. b, An optimised Dox-inducible dCas9-SunTag-VP64 CRISPRa system for enhancer and promoter activation in hESCs (also see Extended Data Fig. 6a). After stable integration of the dox-inducible CRISPRa transgene and the plasmid encoding enhancer/promoter targeting sgRNAs to the genome, hESCs were treated with dox for 48h. GFP-positive cells which expresses the CRISPRa components were subjected to RT-qPCR and immunofluorescence analysis. c, Induction of *SOX17*, *TFAP2C* and *PRDM1* mRNA following CRISPRa of enhancers and/or promoters. Stable hESCs harbouring the CRISPRa transgene and the indicated sgRNA combinations were treated with Dox for 2 days. GFP-positive cells (expressing dCas9-Suntag and scFV-sfgFP-VP64) were isolated for RT-qPCR. Average of 3 biological replicates, with individual replicates shown as data points d-f, Immunofluorescence showing the induction of SOX17 (d), TFAP2C (e) and PRDM1 (f) protein by CRISPRa in hESC lines after 2 days Dox treatment. Experiment was repeated independently for 3 times with similar results.

To scrutinise the most upstream epigenetic events driving the acquisition of hPGC fate, we investigated seven high-confidence putative active enhancers (3 each for *SOX17* and *TFAP2C*, and 1 for *PRDM1*), which gained H3K27ac and lost H3K27me3 during the PreME to hPGCLCs transition. Using a re-engineered Dox-inducible CRISPR activation (CRISPRa) system^54, 55,56^ (Fig. 5a,b, Extended Data Fig. 6a,b, Methods). Independent activation of *SOX17* enhancers 1 and 2 in hESCs modestly induced *SOX17* after 48h, while co-activation of all enhancers led to >10,000-fold upregulation of *SOX17* mRNA compared to non-targeting single guide RNAs (sgRNAs), and the expression of SOX17 protein (Fig. 5c,d). Targeting CRISPRa to a nearby neutral region lacking enhancer chromatin features did not affect *SOX17* expression (Fig. 5c). Importantly, co-activation of promoter and enhancers resulted in *SOX17* upregulation by ~60,000-fold. Similarly, co-activation of all three *TFAP2C* enhancers was sufficient to upregulate *TFAP2C* mRNA and protein, and the activation of the promoter also led to additional upregulation of *TFAP2C* (Fig. 5c,e). Activation of the *PRDM1* promoter alone upregulated *PRDM1* mRNA and protein, with the putative enhancer playing a minor role (Fig. 5c,f). To confirm the context-dependent response of our CRISPRa system, we tested the CRISPRa in HEK293 cells, where the enhancers and promoters of *SOX17* are in a neutral state (Extended Data Fig. 6c-e). Accordingly, targeting *SOX17* regulatory elements in HEK293 cells failed to upregulate *SOX17*, suggesting that the *SOX17* enhancers in hESCs are in a primed/poised epigenetic state.

**Fig. 6:**
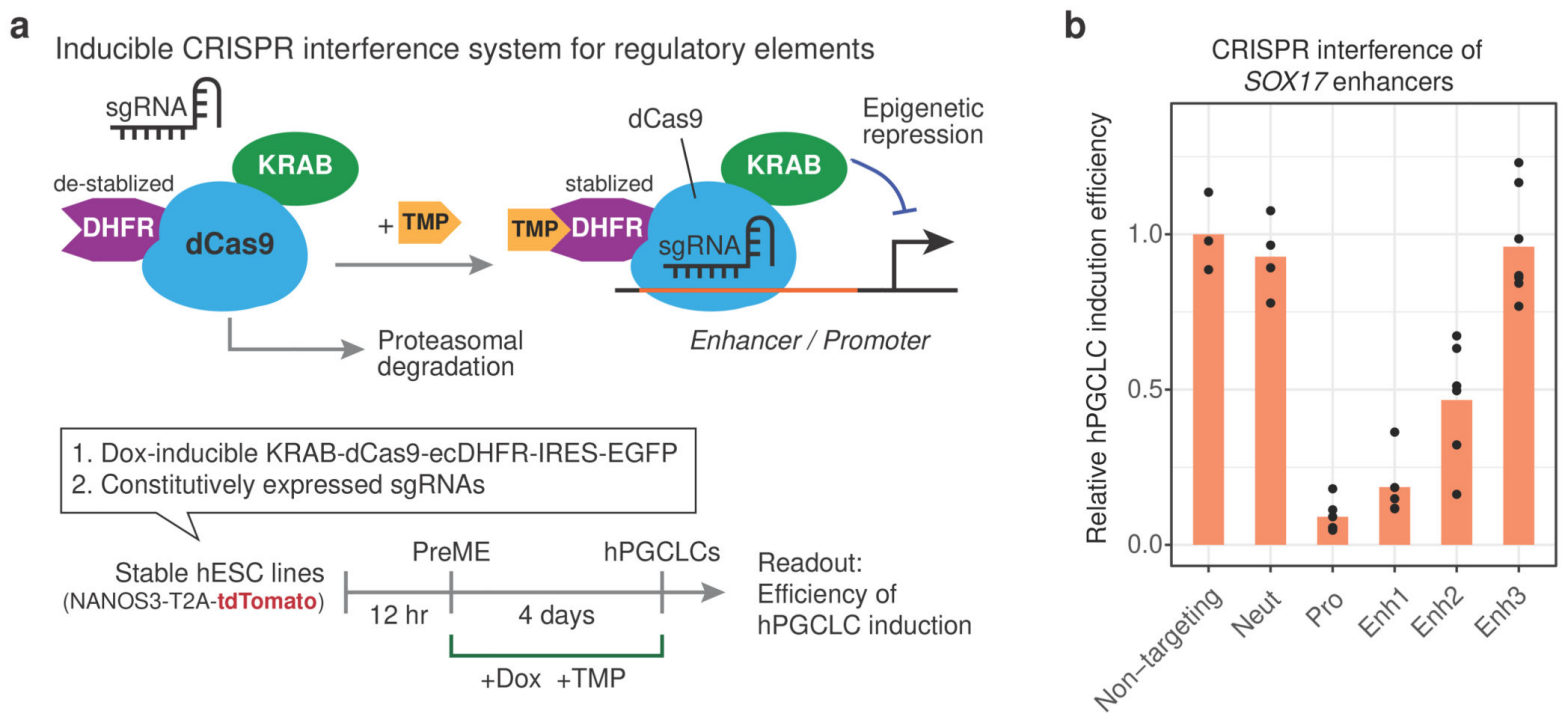
Repression of *SOX17* enhancers by CRISPR interference hampers hPGC specification. a, An inducible CRISPR interference (CRISPRi) system for enhancer repression. A KRAB-dCas9-DHFR transgene was under the control of a Dox-inducible promoter. In the absence of Dox and trimethoprim (TMP; the stabilising DHFR ligand), the DHFR degron causes degradation of KRAB-dCas9-DHFR fusion protein resulted from any leaky activity of the Dox-inducible promoter. Addition of Dox and TMP allow robust mRNA expression and stabilization of the KRAB-dCas9 CRISPR interference machinery, respectively. After stable integration of the inducible CRISPRi transgene and the plasmid encoding enhancer/promoter targeting sgRNAs to the genome, hESCs were induced into PreME and then into hPGCLCs in the presence of Dox and TMP. hPGCLC induction efficiency were evaluated by NANOS3-tdTomato and PDPN expression at d4. b, Reduction in hPGCLC induction efficiency after CRISPRi of *SOX17* enhancers and promoter compared to non-targeting control. Bar plot represents the average relative efficiency, with individual biological replicates shown as data points (non-targeting n=3, neut n=4, pro n=6, enh1 n=5, enh2 n=6, enh3 n=7). Note that targeting of neutral region did not reduce hPGCLC induction.

To test the impact of the repression of the *cis*-regulatory elements in hPGC specification, we engineered a piggyBAC-based inducible CRISPR interference (CRISPRi) plasmid system^57^ (see Fig. 6a, Extended Data Fig. 6f). We generated stable hESC lines bearing sgRNA and Dox-inducible CRISPRi transgenes and found that the repression of *SOX17* promoter alone resulted in >80% reduction of hPGCLC induction efficiency. In comparison, repression of enhancers 1 and 2 resulted in a decrease of 60-75% (Fig. 6b), confirming their critical regulatory activity in hPGC specification.

### CRISPRa-mediated TF induction can drive hPGCLC specification

**Extended Data Fig. 7:**
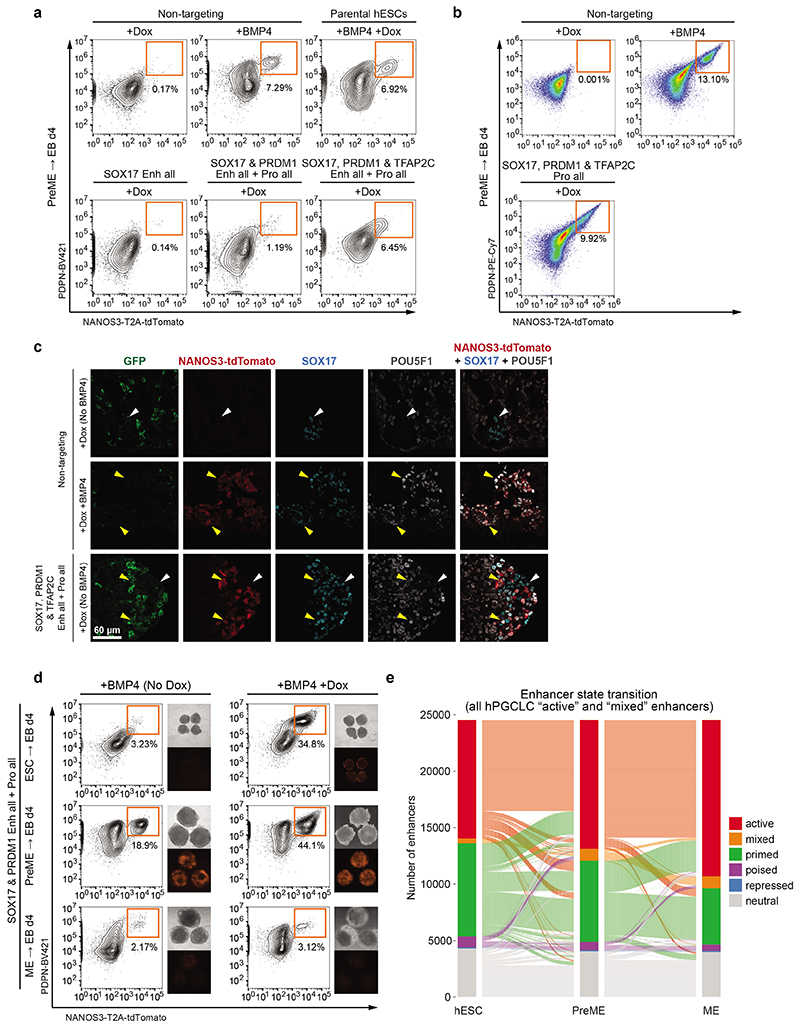
Characterisation of CRISPRa-induced hPGCLCs. a and b, FACS analysis of day 4 EBs generated from PreME of hESC lines harbouring the Dox-inducible CRISPRa transgene with the indicated sgRNA combinations. c, Immunofluorescence showing the co-expression of hPGCLC markers NANOS3-tdTomato, POU5F1 and SOX17 in hPGCLCs (yellow arrowheads) induced by CRISPRa in the absence of BMP4. White arrowheads indicate SOX17 single-positive cells (presumably DE). Representative results of 3 biological replicates. d, Induction of hPGCLCs from hESCs, PreME and ME with or without CRISPR-mediated activation of *SOX17* and *PRDM1* enhancers and promoters. FACS analysis of day 4 EBs shows that the co-activation of *SOX17* and *PRDM1* act synergistically with BMP4 to increase the efficiency of hPGCLC induction from hESCs and PreME, but not from ME. The appearance of the EBs under brightfield and tdTomato fluorescence filter are shown next to the corresponding FACS plots. Representative results of 3 independent experiments. e, Alluvial plots showing enhancer state transitions of hPGCLC-active enhancers in hESCs, PreME and ME.

**Fig. 7:**
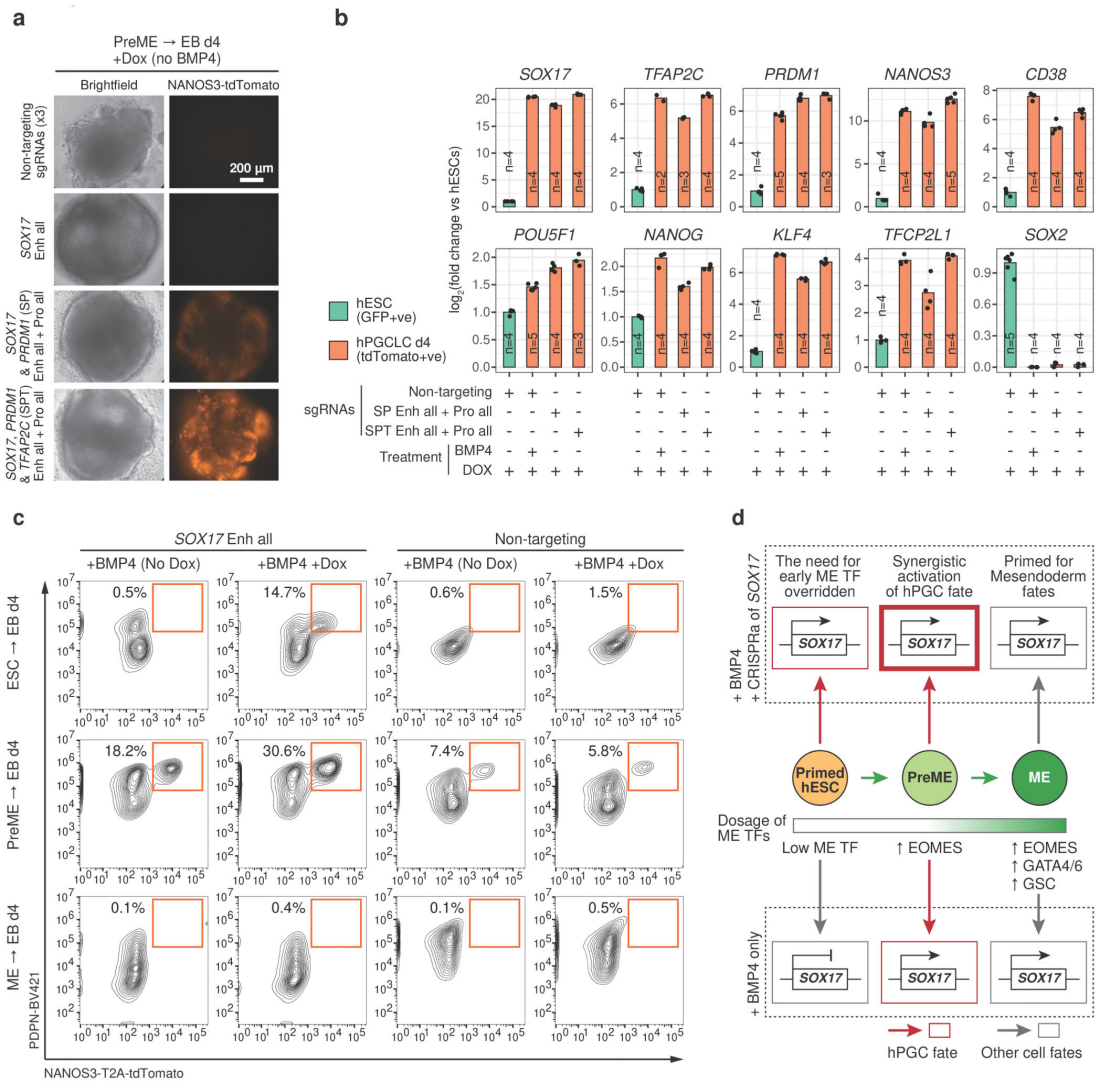
Induction of hPGCLCs by CRISPR activation of key *cis*-regulatory elements. a, Generation of day 4 embryoid bodies from hESC lines harbouring the Dox-inducible CRISPRa transgene with the indicated sgRNA combinations. Note that co-activation of (1) *SOX17* and *PRDM1*; or (2) *TFAP2C*, *SOX17* and *PRDM1*, *cis*-regulatory elements led to the formation of NANOS3-tdTomato-positive hPGCLCs in the absence of BMP4. Experiment was repeated independently for 3 times with similar results. b, Validation of CRISPRa-induced hPGCLCs by RT-qPCR of key hPGC genes. Average of technical replicates, with individual replicates shown as data points and number of replicates indicated in the figure. PCR was replicated 3 times with similar results. c, Induction of hPGCLCs from hESCs, PreME and ME with or without activation of *SOX17* enhancers. FACS analysis of day 4 EBs shows that the activation of *SOX17* enhancers and the addition of BMP4 synergistically increased the efficiency of hPGCLC induction from hESCs and PreME, but not from ME. Orange boxes gate PDPN- and NANOS3-positive hPGCLCs. d, A model elucidating the key role of *SOX17* enhancers in human germline competence.

Next, we tested the sufficiency of the *cis*-regulatory elements for germline commitment. Strikingly, combined CRISPR-mediated activation of *SOX17*, *TFAP2C* and *PRDM1* promoters only or combined with their enhancers was sufficient to induce hPGCLCs from PreME cells without BMP4 (Fig. 7a, Extended Data Fig.7a,b). Comparison between CRISPRa- and BMP4-induced hPGCLCs confirmed activation of target TFs to endogenous levels with a regular expression of early germ cell genes including *NANOS3*, *CD38*, *POU5F1*, *NANOG*, *KLF4* and *TFCP2L1*, and *SOX2* repression (Fig. 7b, Extended Data Fig.7c). Furthermore, co-activation of *SOX17* and *PRDM1* enhancers and promoters also induced hPGC fate without exogenous BMP4 (Fig. 7a, Extended Data Fig. 7a,d), resulting in the upregulation of *TFAP2C* and the establishment of the core hPGC TF network (Fig. 7b, Extended Data Fig. 6b). Our results demonstrate metazoan germline establishment through *cis*-regulatory element activation.

### Sequential enhancer activation defines germline competence

One hypothesis for the transient gain of germline competence in PreME was that the *cis*-regulatory elements of hPGC specifiers became transiently primed/poised for activation. Surprisingly, the enhancers and promoters of *SOX17*, *PRDM1* and *TFAP2C* were already in primed or poised state (marked by H3K4me1 with or without H3K27me3) in hESCs and remained so in PreME and ME (Fig. 5a). Indeed, >80% of hPGCLC active enhancers are similarly in active, primed, or poised states in hESCs, PreME and ME (Extended Data Fig. 7e), including the enhancers of key hPGC genes *POU5F1*, *NANOG* and *NANOS3* (Fig. 4d).

Since there is no SOX17 upregulation or hPGCLCs induction in hESC and ME in response to BMP4^9^, we asked if activation of *SOX17* enhancers allows induction of hPGCLCs from hESCs. Notably, activation of *SOX17* enhancers by CRISPRa in conjunction with BMP induced hPGCLCs specification from hESCs, which was not observed with non-targeting sgRNAs (Fig. 7c). Moreover, the activation of *SOX17* enhancers and the addition of BMP4 in PreME had synergistic effects with a doubling of the efficiency of hPGCLC induction compared to BMP4 treatment alone. Consequently, the gain of competence in PreME from hESCs might be attributed to a permissive TF combination that can activate *SOX17* enhancers (Fig. 7d).

**Fig. 8:**
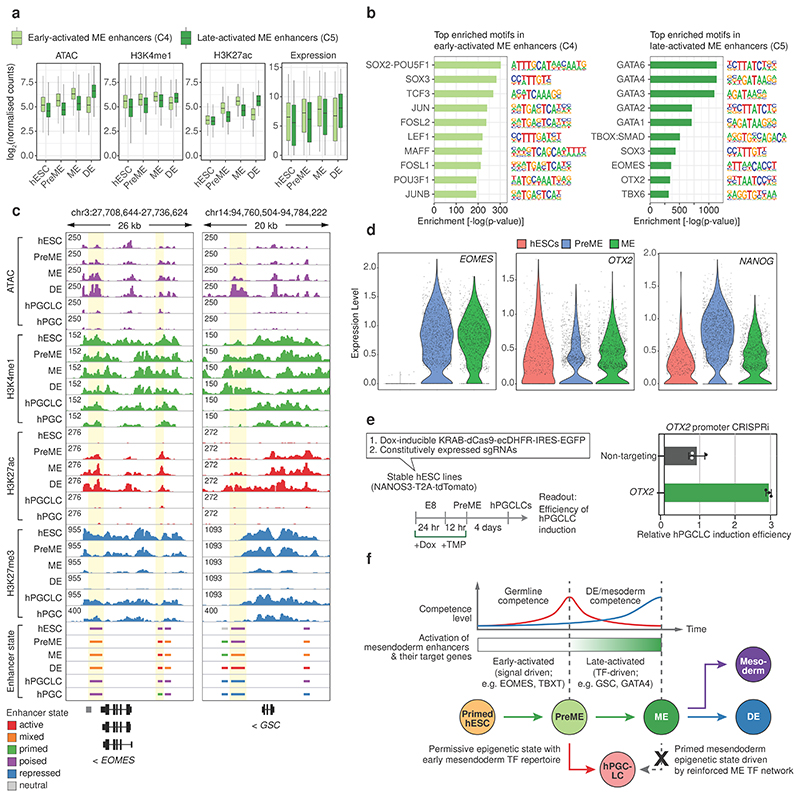
Sequential activation of mesendodem and germline enhancers explains germline competence. a, Boxplots of ATAC, H3K4me1 and H3K27ac signals in early-activated (C4) and late-activated (C5) mesendoderm enhancers and the expression levels of the associated high confidence target genes during mesendoderm differentiation. Box plots depict the median, lower and upper hinges correspond to the 25th and 75th percentiles, whiskers correspond to 1.5 x inter-quartile range from the hinges. C4 = 1,909 enhancer and 209 associated genes; C5 = 3,703 enhancers and 372 associated genes. b, Top ten TF motifs enriched in early-activated and late-activated mesendoderm enhancers. c, Genome browser snapshots showing the early-activated enhancer of *EOMES* and the late-activated enhancer of *GSC* (highlighted). For simplicity, only enhancers that were assigned to gene with high confidence (Extended Data Fig. 2e,f) are shown. d, Violine plots summarizing expression levels of the indicated genes in individual cells in the hESC, PreME and ME state analysed by scRNA-seq. e, Experimental design of inducible CRISPRi mediated *OTX2* knockdown in PreME cells (left) and bar chart depicting the PGCLC specification efficiencies of control and PreME cells expressing gRNAs to target CRISPRi to the two *OTX2* promoters (right). Width of the bar plot represent the mean of the replicates. Error bars represent S.D. of 3 biological replicates (shown as data points). f, A model explaining the transient gain and subsequent loss of human germline competence during the epigenetic priming of hESCs to ME.

Next, we considered enhancers dynamically activated during mesendoderm differentiation, designated as ‘early’ (C4) and ‘late’ (C5) mesendoderm enhancers (Fig. 2c,d). Early mesendoderm enhancers (C4) lacked H3K27ac and were relatively inaccessible in hESCs but became increasingly opened up and gained H3K27ac in PreME and ME (Fig. 8a). The high confidence targets of these enhancers were involved in ‘Wnt signalling pathway’ and ‘mesoderm formation’ (Fig. 2d), including *EOMES*, which is necessary for SOX17 upregulation during hPGCLC specification^20, 58^. Motif enrichment analysis suggested that early mesendoderm enhancers were activated by downstream mediators of the FGF (JUN, FOS) and canonical WNT signalling pathway (LEF1, TCF3, TCF7L2)^59^ (Fig. 8b). Indeed, *EOMES* is a known downstream target of the WNT signalling pathway^60^. On the other hand, late activated mesendoderm enhancers (C5) only became accessible and enriched for H3K27ac in ME, with further chromatin opening and activation in DE (Fig. 8a). These enhancers targeted master mesoderm and endoderm regulators (*GSC*, *GATA4*, *CER1* and *LHX1*) and were enriched for GATA motifs, coinciding with *GATA4* and *GATA6* upregulation in ME and DE (Fig. 2e, 8b,c). Notably, the OTX2 motif was enriched explicitly in late activated mesendoderm enhancers.

**Extended Data Fig. 8:**
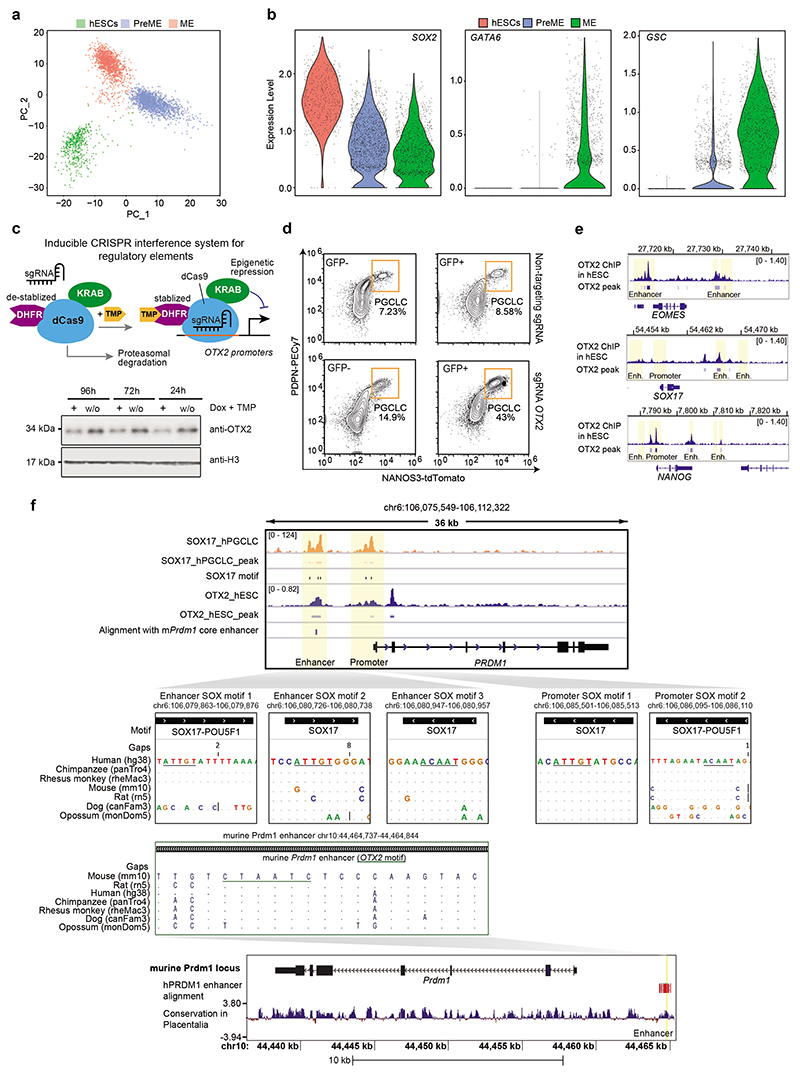
Sequence conservation of the human *PRDM1* regulatory element. a, PCA analysis scRNA-seq profiles of cells in the hESC, PreME and ME state b, Violin plots summarizing expression levels of the indicated genes in individual cells in the hESC, PreME and ME state analysed by scRNA-seq. c, Schematics of the inducible CRISPR interference (CRISPRi) system used to repress the two *OTX2* promoters (upper panel). Western Blots depicting OTX2 and H3 levels in transgenic hESCs treated with vehicle or Doxcycline and TMP for the indicated time periods (lower panel). Molecular weights of marker proteins are depicted in kilodaltons (kDa). Representative experiment, knockdown efficiency tested two times independently at the shown timepoints. d, FACS analysis of PGCLCs expressing non-targeting or sgRNA targeting the *OTX2* promoters in the presence or absence of KRAB-dCas9-ecDHFR (GFP). Representative experiment out of 3 independent technical replicates shown in Fig. 8e. e, Genome browser snapshots showing OTX2 ChIP-seq signals and peaks in hESCs (GSE61475)^64^. Enhancer identified in this work are indicated in yellow. f, Upper panels: BLAT alignment of the core murine *PRDM1* enhancer (B108)^72^ to the human genome. Conservation of the SOX motifs in the putative enhancer and promoter of human *PRDM1* across seven mammalian species. MULTIZ whole-genome alignment showed that 4 out of 5 core SOX motifs (‘ATTGT’, underlined) in the human *PRDM1* enhancer and promoter are conserved in mice. Grey dot indicates exact match. Blank space represents absence of the corresponding sequence in the indicated species. Lower panels: BLAT alignment of the human *PRDM1* enhancer to the murine genome showing the conservation of the OTX2 motif in the murine *PRDM1* enhancer^72^.

Next, we analysed the cellular heterogeneity of hESCs, PreME and ME by single-cell RNA sequencing (scRNA-seq), revealing that these cell types represent distinct transcriptomic states without clear subpopulations (Extended Data Fig. 8a). However, individual genes, including *EOMES* and *OTX2*, exhibit heterogeneous expression (Fig. 8d, Extended Data Fig. 8b). In many PreME cells, the *OTX2* expression level was reduced compared to hESCs and ME, while *EOMES* expression increased strongly relative to hESCs. We used our inducible CRISPRi system to test whether a further reduction of *OTX2* in PreME could promote PGCLC specification, and indeed there was a substantial gain of PGCLC specification efficiency (Fig. 8e and Extended Data Fig. 8c,d).

Therefore, the temporal reduction of *OTX2* expression in PreME cells exhibiting increasing *EOMES* levels might critically define the gain of germline competence in the absence of later activated mesendoderm TFs, e.g., GSC, GATA6. High levels of OTX2 and other mesendoderm TFs in ME abrogate germline competence and promote somatic fates (Fig. 8f).

## Discussion

We demonstrate how an integrated signalling response manifests in altered epigenetic states, and the activation of developmental TFs drives human germline-soma segregation (Fig. 8f). During the hESCs-PreME transition, endogenous FGF and WNT signalling^15, 61^ (Fig. 1a) activate early mesendoderm enhancers and genes, including *EOMES* required for hPGC specification^20^. WNT signalling and elevated NANOG expression in response to NODAL signalling likely contribute to the transient OTX2 reduction in a subset of PreME cells, conferring germline competence^62^ while delaying the mesendodermal fate. A reciprocal OTX2-NANOG relationship has been reported in human blastocysts and neuronal differentiation^62, 63^. Consistently, CRISPRi mediated *OTX2* knockdown promotes PGCLC competence Cell-type-specific functions of OTX2 are possible throughout hESCs to ME transition^64^, following redistribution and altered chromatin interactions^64^ (Fig.8b); binding to regulatory elements in hESCs might repress hPGCLC specification (Extended Data Fig. 8e). In mice, OTX2 also restricts germline competence by interfering with TFs that drive murine PGC fate while promoting a primed pluripotent state which lacks germline competence^65, 66^.

During the PreME-to-ME transition, early mesendoderm TFs and ACTIVIN-SMAD signalling induce expression of genes like *GSC*, *OTX2*, and *GATA4*^67, 68^, which in turn, activate somatic enhancers in ME that profoundly change the cellular response to BMP and SOX17 and drive the cells past the ‘point of no return’ for the hPGC fate (Fig. 7d and 8f). Only a fraction of epiblast cells commits to the germ cell lineage in mouse and pig embryos, indicating a high cell-intrinsic barrier for PGC fate^6, 9^. Similarly, only 10-40% of PreME cells differentiate into hPGCLCs *in vitro*, suggesting that only cells with the appropriate epigenetic state, mesendoderm TF dosage, and cell cycle stage^69^, might commit to the hPGC fate.

The high hPGCLC specification upon CRISPR-mediated *SOX17* enhancer activation suggests that *SOX17* transcriptional induction represents an essential barrier for hPGC specification. A permissive epigenetic state of the *SOX17 cis*-regulatory elements is a component of germline competence (Fig. 7c). The oncogenic transformation of hPGCs into pluripotent embryonal carcinoma (EC) cells and germ cell tumours entails the loss of SOX17 and the gain of SOX2 function. Therefore, the epigenetic status of the regulatory elements is likely of clinical relevance^22, 70^.

EOMES is essential for germline competence, yet additional TFs are probably required for *SOX17* induction since only a fraction of EOMES-positive PreME cells acquire the hPGCLC fate^9, 16, 20, 58^. BMP4 signalling is unlikely sufficient for *SOX17* induction since the expression of BMP-responsive genes *ID1*, *ID2*, and *MSX2*, precedes *SOX17* substantially ^71^. Putative TF binding sites within the *SOX17* enhancers, including POU5F1, EOMES, GATA3, TFAP2A/C and SMAD1, suggests a combinatorial and cooperative action of TFs at individual enhancers to drive *SOX17* expression beyond a threshold for hPGC specification. SOX17 and TFAP2C activate germline enhancers and cooperate with their direct downstream targets to sculpt the epigenome for hPGC fate. Remarkably, CRISPR-mediated activation of the *cis*-regulators of *SOX17*, *TFAP2C* and *PRDM1* is sufficient for hPGCLC induction without BMP4.

During hPGCLC specification, *PRDM1* is a direct target of SOX17 but not in mice. Despite the mouse-human differences, the human *PRDM1* enhancer bears a substantial resemblance to the murine counterpart, which interacts with OTX2 during retina development^72^. Since the OTX2-binding motif is conserved in the human *PRDM1* enhancer, OTX2 may modulate *PRDM1* expression. Since the human and mouse *PRDM1* loci show conservation of four out of five SOX motifs in their enhancers and promoters (Extended Data Fig. 8f), SOX17 can likely regulate mouse *PRDM1* as exemplified by their co-expression in mouse visceral endoderm^12, 73, 74^. Altogether, SOX17 is the critical regulator of hPGC fate, while PRDM1, PRDM14, and potentially SOX2 fulfil this role in mice^8, 17, 75^.

Regulatory elements of TFs defining germ cell identity, e.g., *SOX17* and *TFAP2C*, are active in nascent hPGCLCs and more advanced gonadal hPGCs (Fig. 2c, cluster C9). During hPGC maturation towards gametogenesis, genes regulating migration, epigenetic resetting, meiotic entry, and genome defence become transcriptionally induced with the activation of the associated regulatory elements^26^. While hPGCLCs co-cultured with mouse gonadal tissue can develop an oogonia-like state, the process is highly inefficiently (~1%) and requires four months of culture^76, 77^. Investigating the regulatory elements in hPGCLCs and hPGCs could help optimise hPGCLC differentiation conditions by determining likely roadblocks that hinder maturation. Our re-designed CRISPRa and CRISPRi systems that allow efficient multiplexed modulation of *cis*-regulatory elements could be deployed to discover and overcome epigenetic obstacles during the development of hPGCLC towards gametogenesis.

The origin of hPGCs during peri-implantation development remains a challenge, with the posterior epiblast and nascent amnion being possible sites of PGC specification^10^. In a rare human gastrulating embryo, hPGCs were found in the epiblast^13^. In some mammalian embryos that develop as bilaminar discs as in humans, PGCs originate in the posterior epiblast^78, 79^. In the future, comparing the epigenetic profiles of PreME or hPGCLCs with amniotic ectoderm-like cells^80^ might help to determine similarities between these cells.

With the epigenetic principles of human germline competence, specification, and development, we establish a framework for in vitro gametogenesis and for decoding the mechanisms promoting the critical epigenetic resetting in the germline for totipotency and its evolutionary divergence amongst mammals. Understanding germline networks will help to explore the pathogenesis of infertility, germ cell cancer and age-related diseases of somatic tissues that lack the unique epigenetic resetting event present in the ‘immortal’ germline.

## Methods

### Ethics Statement

Human embryonic tissues were used under permission from National Health Service Research Ethical Committee, UK (Research Ethics Committee number: 96/085). Patients (who had already decided to undergo the termination of pregnancy operation) fully and freely consented to donate the foetal tissues for medical and academic research. Medical or surgical termination of pregnancy was carried out at Addenbrooke's Hospital, Cambridge, UK.

### Collection of hPGCs from human embryos

Crown-rump length, anatomical features, including limb and digit development, was used to determine the developmental stage of human embryos with reference to Carnegie staging (CS). The sex of embryos was determined by sex determination PCR as previously described^82^. Human embryonic genital ridges from individual male embryos (wk7-9) were dissected in PBS and separated from surrounding mesonephric tissues. The embryonic tissues were dissociated with Collagenase IV (Sigma, C5138) and DNase I in DMEM-F/12 (Gibco) at 37°C for 15-30 minutes (depending on tissue size). Cell suspension was diluted with FACS medium (PBS with 3% foetal bovine serum & 5 mM EDTA) and centrifuged at 500 xg for 5 minutes. The cell pellet was suspended with FACS medium and incubated with Alexa Fluor 488-conjugated anti-alkaline phosphatase (BD Pharmingen 561495, 5 ul) and APC-conjugated anti-c-KIT (Invitrogen CD11705, 5ul) antibodies for 20 minutes at room temperature in the dark. Cells were spun down, resuspended in FACS medium and passed through a 35 μm cell strainer. FACS was performed with SH800Z Cell Sorter (Sony), and FACS plots were generated by FlowJo (10.7.1). The alkaline phosphatase- and cKIT- double-positive populations were sorted onto Poly-L-Lysine Slides (Thermo Fisher Scientific) and fixed in 4% PFA. Alkaline phosphatase staining was performed with Leukocyte Alkaline Phosphatase Kit (Sigma) to determine the purity of hPGCs. Only samples with >97% purity were used for epigenomic analysis.

### Human ESC culture, differentiation and collection

NANOS3–tdTomato reporter hESCs (WT), NANOS3–tdTomato hESCs bearing Dex-inducible *SOX17* and Dox-inducible *PRDM1* transgenes (WT + iSOX17 + iPRDM1) were established previously^9^. All cell lines were confirmed as mycoplasma negative. hESCs were maintained on vitronectin-coated plates in Essential 8 medium (Thermo Fisher Scientific) according to the manufacturer’s protocol. Cells were passed every 3-5 days using 0.5 mM EDTA in PBS as small cell clumps.

ME, hPGCLC and DE were induced from NANOS3–tdTomato reporter hESCs^9^ using the aRB27 basal medium, which was composed of Advanced RPMI 1640 Medium (Thermo Fisher Scientific) supplemented with 1% B27 supplement (Thermo Fisher Scientific), 0.1 mM NEAA, 100 U/ml penicillin, 0.1 mg/ml streptomycin, 2 mM L-glutamine. To induce mesendoderm, trypsinised hESCs were seeded on a vitronectin-coated dish at 200,000 cells per well in a 12-well plate and cultured in mesendoderm induction medium for 12h (PreME) and 24h (ME). Mesendoderm induction medium contained aRB27 medium supplemented with 100 ng/ml activin A (Department of Biochemistry, University of Cambridge), 3 μM GSK3i (Miltenyi Biotec) and 10 μM of ROCKi (Y-27632, Tocris bioscience). To induce DE from ME, mesendoderm induction medium was replaced with a DE induction medium after washing with PBS once, and cells were cultured for a further 2 days. DE induction medium was composed of aRB27 medium supplemented with 100 ng/ ml activin A (Department of Biochemistry) and 0.5 μM BMPi (LDN193189, Sigma). To induce hPGCLCs, PreME cells were trypsinised and plated into Corning Costar Ultra-Low attachment multiwell 96-well plate (Sigma) at 4,000 cells per well in hPGCLC induction medium, which composed of aRB27 medium supplemented with 500 ng/ml BMP4,10 ng/ml human LIF (Department of Biochemistry), 100 ng/ml SCF (R&D systems), 50 ng/ml EGF (R&D Systems), 10 μM ROCKi, and 0.25% (v/v) poly-vinyl alcohol (Sigma). Cells were cultured as floating aggregate for 2-4 days.

For ATAC-seq, RNA-seq and ChIP-seq, hESCs, PreME, ME, DE, hPGCLCs were collected from two independent series of induction experiments. hESCs, PreME and ME were trypsinised with 0.25% trypsin/EDTA and subjected to FACS and gated for NANOS3-tdTomato negativity. Day 2 DE was stained with PerCP-Cy5.5 conjugated anti-CXCR4 antibody (BioLegend 306516, 5 ul/million (M) cells) and CXCR4-positive DE cells were collected. For hPGCLCs, day 2 and day 4 embryoid bodies (EBs) were trypsinised with 0.25% trypsin/EDTA at 37°C for 15 min. hPGCLCs were sorted using the highly specific PGC marker, NANOS3-tdTomato.

To study the transcriptional response after SOX17 or PRDM1 overexpression, PreME were first induced from NANOS3–tdTomato hESCs bearing Dex-inducible *SOX17* and Dox-inducible *PRDM1* transgenes (WT + iSOX17 + iPRDM1). PreME aggregates were treated with vehicle (water), 100 μM dexamethasone (Sigma) or 0.5 μg/ ml doxycycline (Sigma) in the absence of cytokines. Aggregates were harvested for total RNA extraction 12h after transgene induction.

Two biological replicates were collected for each transcriptome and epigenome analysis.

### Generation of RNA-seq libraries

hESCs, PreME, ME, DE, hPGCLCs and hPGCs were sorted directly into extraction buffer of PicoPure RNA Isolation Kit (Applied Biosystems) and RNA was extracted according to manufacturer’s protocol with on-column DNase I treatment (Qiagen 79254). RNA-seq libraries were generated from 5 ng total RNA using Ovation RNA-Seq System V2 (Nugen) and Ovation Rapid DR Multiplex System (Nugen)^25^. Libraries were quantified by qPCR using KAPA Library Quantification Kit (Kapa Biosystems) using QuantStudio 6 Flex Real-Time PCR System (Applied Biosystems) and validated using Agilent TapeStation 2200 with High Sensitivity D1000 ScreenTape. Libraries were subjected to single-end 50 bp sequencing on HiSeq 4000 sequencing system (Illumina), resulting in >30 millions single-end reads per sample.

RNA-seq libraries of PreME aggregate with SOX17 or PRDM1 overexpression were generated by the NEBNext Ultra II Directional RNA Library Prep Kit for Illumina (NEB, E7760S) and the NEBNext Poly(A) mRNA Magnetic Isolation Module (NEB, E7490) according to manufacturer’s protocol. Quantified and validated libraries were subjected to single-end sequencing on HiSeq 4000 sequencing system (Illumina).

### Generation of ATAC-seq libraries

Cells were sorted directly into Nuclei EZ Storage Buffer (Sigma, NUC-101) and stored at -80°C. ATAC-seq libraries were prepared following the Omni-ATAC protocol described by Corces et al.^27^ with the following modifications: Tagmented DNA was amplified using the KAPA HiFi HotStart Real-Time Library Amp Kit (Roche) with modified Nextera dual indexed primers as listed in Supplementary Table 7. Amplified libraries were purified using Ampure XP beads (Beckman Coulter) with double-sided size selection (1^st^ bead selection: 0.5x; 2^nd^ bead selection: 1.2x) according to manufacturer’s protocol. Quantified and validated libraries (~150-1000 bp) were subjected to pair-end sequencing on HiSeq 4000 sequencing system (Illumina), resulting in >30 millions single end reads per sample.

### Generation of chromatin ChIP-seq libraries

Histone modification ULI-NChIP-seq was conducted as described in Brind'Amour et al.^28^. In brief, cells were FACS sorted in 3% FCS/PBS, pelleted by centrifugation, and stored in 20 μl Nuclei EZ Storage Buffer at -80°C. Cells were thawed on ice, incubated with 2 μl of 1% Triton X-100, 1% Sodium deoxycholate and digested with Micrococcal Nuclease (MNase) (NEB). MNase activity was blocked by addition of 11 μl 100 mM EDTA and cell lysate was incubated for 1h in 400 μl of IP buffer at 4°C followed by 2h incubation in the presence of 5 μl blocked protein A/G beads (blocking buffer: 100 μg/ml yeast tRNA, 0.1% BSA in IP buffer). After the removal of the protein A/G beads, the pre-cleared cell lysate was added to the antibody (Supplementary Table 7) bead complex (antibody was incubated with 5 μl blocked protein A/G beads for 3 hour on 4°C) overnight at 4°C. Unbound chromatin was removed, and beads were sequentially washed for 4 min for 1.) two times with low salt wash buffer, 2.) two times with high salt buffer, and 3.) two times with LiCl wash buffer (20 mM Tris-HCl pH 8.0, 2 mM EDTA, 250 mM LiCl, 1% NP-40, 1% Sodium deoxycholate). To elute the bound DNA, beads were incubated in Proteinase K digestion buffer (20 mM HEPES pH 8.0, 1 mM EDTA, 0.5% SDS, 1 mg/ml RNase, 0.4 mg/ml Proteinase K) for 15 min at 55°C and 1h at 65°C. The DNA was purified from the eluate through AMPure XP beads and eluted in 20 μl EB buffer (MinElute Reaction Cleanup Kit; Qiagen). ULI-NChIP-seq libraries were generated by the KAPA Hyper Prep Kit (KAPA Biosystems) according to manufacturer’s protocol. To minimize adaptor dimer formation, the NEBNext Adaptor and NEBNext Index PCR Primers from the NEBNext® Multiplex Oligos for Illumina (Index Primers Set 1) (NEB, E7335S) were used. After library amplification, libraries were purified by AMPure XP beads with double-sided size selection as for ATAC-seq libraries. Quantified and validated libraries were subjected to paired-end sequencing on HiSeq 4000 sequencing system (Illumina), resulting in 27-96 millions paired end reads per sample. All histone modification antibodies used in this study (Supplementary Table 7) were extensively validated for their sensitivity and specificity by ULI-NChIP qPCR and ULI-NChIP-seq.

### Generation of transcription factor ChIP-seq libraries

For HA-SOX17 and myc-PRDM1 ChIP-seq, PreME cells were induced from NANOS3– tdTomato hESCs bearing Dex-inducible *SOX17* and Dox-inducible *PRDM1* transgenes (WT + iSOX17 + iPRDM1). Subsequently, hPGCLCs were induced by hPGCLC induction medium in the presence of 100 μM dexamethasone (Sigma) (iSOX17) or 0.5 μg/ ml doxycycline (Sigma) (iPRDM1). For HA-SOX17 ChIP-seq in DE, ME cells were induced from the same hESC line, followed by DE induction in DE medium supplemented with 100 μM dexamethasone (iSOX17). The whole day 2 embryoid bodies with hPGCLCs and day 2 DE cells (around 1.5-1.7 million cells) were collected for chromatin immunoprecipitation using the SimpleChIP Enzymatic Chromatin IP Kit (Magnetic Beads) (Cell Signaling Technology, 9003)^40^. Briefly, the cell pellets were washed twice with cold PBS containing 0.1% BSA and then fixed with paraformaldehyde. Following chromatin digestion with MNase, 2% volume of nuclei lysate was removed and stored at -80°C as input control while the rest of the lysate was subjected to immunoprecipitation with anti-HA (Cell Signaling Technology, 3724) or anti-Myc (Cell Signaling Technology, 2276) antibody. After elution of chromatin, reversal of cross-links and DNA purification, the ChIP and input DNA were prepared for sequencing using the KAPA HyperPrep Kit following the manufacturer’s instructions. Quantified and validated libraries were subjected to single- end or paired-end sequencing on HiSeq 4000 sequencing system (Illumina).

### RNA-seq data processing

For non-directional RNA-seq libraries listed in Extended Data Fig. 1b and 1c, libraries were checked by *FastQC*(v0.11.5)^83^. The low-quality reads and adaptor sequences were removed by *Trim Galore*(v0.4.1)^84^ using the default parameters. The pre-processed reads were mapped to the human reference genome (UCSC GRCh38/hg38) using *STAR*(2.7.1a)^85^ (parameters: ‘*-- outFilterMismatchNoverLmax 0.05 --outFilterMultimapNmax 50 --outMultimapperOrder Random*’) guided by the Gencode Human Release 30 comprehensive gene annotation^86^. Raw read counts per gene were extracted by the *featureCounts* function of the Subread package(1.6.2) using the default parameters. Normalized read counts and differentially expressed genes (absolute(log_2_(fold change)) >2 and adjusted p-value <0.05) were obtained using *DEseq2*(1.26.0) in R(3.6.2)/*Bioconductor*(3.10.1). For all expression analysis, a log_2_(normalized counts +1) transformation was applied. Only ‘protein_coding’ and ‘lincRNA’ genes were retained in subsequent genome-wide analysis. Unsupervised hierarchical clustering (UHC) was performed using the R *hclust* function with the Ward’s method using all expressed genes. All UHC dendrograms in this paper were reordered using the optimal leaf ordering algorithm in the R *cba*(0.2-21). Spearman’s correlation analysis was performed using the R *cor* command, considering the top 25% most variable genes. The accompanying dendrogram was generated using (1 - Spearman’s correlation coefficient) as distance measures.

*SOX17* or *PRDM1* overexpression RNA-seq libraries were processed similarly but with the following modifications at the read counting step: To account for the directional reads: raw read counts per gene were extracted by *featureCounts* with the parameter ‘-s 2’. To exclude exogenous *SOX17* and *PRDM1* transcripts originated from the transgenes, only reads overlapping the 5’ and 3’ untranslated regions (UTRs) of *SOX17* and *PRDM1* transcript isoforms were counted. This allowed the detection of endogenous expression levels of *SOX17* and *PRDM1* in response to ectopic SOX17 and PRDM1.

RNA-seq dataset of *SOX17*, *TFAP2C* and *PRDM1* knockout and control hPGCLCs/aggregates were retrieved from NCBI Gene Expression Omnibus (GSE99350)^20^. Reads were trimmed to 76 bp by *Trimmomatic* (0.39)^87^ and adaptors were trimmed by *cutadapt* (1.15) with options ‘-e 0.1 -q 20 -n 2 -O 1 -m 30 -a CTCGAGGGCGCGCCGGATCC -g CTCGAGGGCGCGCCGGATCC -a AAAAAAAAAAAAAAAAAAAA -a TTTTTTTTTTTTTTTTTTTT’. Trimmed reads were mapped to the human reference genome using *STAR*, counted by featureCounts and normalized by *DEseq2*. Differential expression threshold between knockout and control was set at absolute(log_2_(fold change)) >1 and adjusted p-value <0.05.

### ATAC-seq and chromatin ChIP-seq data processing

Paired-end ATAC-seq reads were quality- and adaptor-trimmed by *Trim Galore* using default parameters. Trimmed reads were mapped to the human reference genome (UCSC GRCh38/hg38) by Bowtie 2 (v2.3.4.1)^88^ with options ‘--local -X 2000 --no-mixed --no-discordant’, hence retaining properly paired reads with a maximum fragment length of 2000 bp. Unmapped reads, non-primary reads, supplementary alignment and quality-control-failed reads were removed using samtools(1.7) *view* with option ‘-F 2828’^89^. Duplicated reads were marked and removed by the MarkDuplicates function in Picard Tools(2.9.4-SNAPSHOT) (Broad Institute). Fragments mapped to hg38 blacklisted regions (http://mitra.stanford.edu/kundaje/akundaje/release/blacklists/hg38-human/hg38.blacklist.bed.gz), non-canonical hg38 contigs and mitochondrial DNA (chrM) were removed. To adjust the read start sites to represent the centre of the transposon binding event, all reads aligning to the positive strand were offset by +4 bp, and all reads aligning to the negative strand were offset -5 bp^32^. For peak calling and generation of bigwig signal tracks, ‘cleaned’ ATAC-seq libraries were subsampled using Picard DownSampleSam so that each library contains approximately the same number of paired-end reads.

For visualization in Integrative Genomics Viewer (IGV) genome browser (2.4.10), the individual downsampled libraries and the merged downsampled libraries of the two replicates (pooled replicates) were converted into signal tracks using deepTools(3.0.2)^90^ *bamCoverage* with fragments per kilo base per million normalization (FPKM) normalization (options: ‘--binSize 10 --normalizeUsing RPKM --ignoreForNormalization chrX chrY --extendReads --samFlagInclude 64’). For simplicity, only the merged signal tracks are shown in the genome browser snapshot figures. Paired-end ULI-NChIP-seq reads were processed using the same pipeline for ATAC-seq, but without adjustment of read position.

### Reproducible peak calling

Prior to peak calling from ATAC-seq libraries, paired-end reads with fragment size <120 bp (nucleosome-free open chromatin) were extracted using deepTools *alignmentSieve* and downsampled. Peaks for ATAC-seq and histone ChIP-seq libraries were called following the Encode replicated peak calling guidelines (https://www.encodeproject.org/pipelines/ENCPL272XAE/)^91^ with modifications to accommodate for paired-end libraries. To obtain peaks with high resolution and confidence, narrow peak call was used for all marks using the input reads as background. Peaks were initially called for each biological replicate (downsampled to the same read depth), for the pooled replicates, and for the pooled pseudoreplicates of each biological replicate using MACS2(2.1.2)^92^ with a relaxed p-value threshold of 0.05 (options: ‘-g 3e9 --keep-dup all -p 0.05’). Each pseudoreplicate consists of half the reads of each biological replicate, chosen at random without replacement. Narrow peaks from the pooled replicate set were retained if they overlapped peaks from both biological replicates or peaks from both pooled pseudoreplicates (20% and 30% overlap by peak length for ATAC peaks and histone peaks, respectively). This peak calling strategy allows for the retention of marginal peaks in one replicate to be rescued by a strong biological replicate. To obtain a final high confidence peak set, the reproducible peaks were further filtered using the MACS2 q-value (false discovery rate <0.0001 for ATAC peaks and <0.001 for histone peaks).

### Analysis of individual epigenomic mark

For each histone mark, a combined peak set of all cell types was generated using *bedtools* (2.26.0) *merge*^93^. Raw read counts at genomic 1 kb tiling bins (BEDOPS (2.4.35)^94^) that overlapped any combined peak were extracted using featureCounts (options: ‘-f -p -O’). Normalized and differential signals at each bin were obtained by *DEseq2* in R using relative read depth between libraries as size factors, followed by log_2_(normalized counts +1) transformation. Dynamic peaks were defined as absolute[log_2_(signal fold change)] >1 and adjusted p-value <0.05 in the sample pairs shown in Extended Data Fig. 2d. ATAC-seq analysis was performed in a similar manner, except that reads were counted using a combined ATAC peak set (instead of 1 kb genomic bins). Spearman’s correlation analysis of replicates was performed using the R *cor* command and the accompanying dendrogram was generated using (1 - Spearman’s correlation coefficient) as distance measures (with optimal leaf ordering). PCA was performed using the R *prcomp* function.

For peak distribution analysis (Extended Data Fig. 1e), distance between the summit of ATAC peaks or the centres of histone modification peaks and the nearest TSS (protein coding and lincRNA genes in the Gencode Human Release 30 basic gene annotation) was extracted using the *annotatePeaks.pl* script of HOMER(v4.10.4)^95^.

### Promoter epigenetic state analysis

Promoter regions were defined as TSS ± 1 kb of all protein-coding and lincRNA transcripts in the Gencode Human Release 30 basic gene annotation (61,594 non-redundant promoters). Meta-gene profile plot and heatmap of histone modification pattern was generated by deepTools *computeMatrix* and *plotHeatmap* with k-means clustering.

We defined promoters as active, mixed, poised, repressed and neutral based on the overlap with H3K4me3, H3K27ac and H3K27me3 peaks (at least 20% overlap by promoter length) in each cell type as depicted in Extended Fata Fig. 3a. Promoters without H3K4me3, H3K27ac or H3K27me3 peaks were defined as ‘neutral’.

To study promoters epigenetic dynamics, read counts of ATAC, H3K4me1, H3K4me3, H3K27ac and H3K27me3 at promoter regions were extracted by featureCounts (options: ‘-f -p -O’ ) and normalized by featureCounts using relative read depth between libraries as size factors. To identify dynamically repressed promoters (Extended Data Fig. 3c), promoters that were ‘mixed’, ‘poised’ or ‘repressed’ in any cell types and exhibited differential H3K27me3 signals (absolute[log_2_(signal fold change)] >1 and adjusted p-value <0.05) were extracted and subjected to k-means clustering using the R *kmeans* function based on z-scores of log_2_(normalized H3K27me3 counts +1) across cell types.

To evaluate the predictive power of chromatin marks at promoter for gene expression by receiver operating characteristic (ROC), non-neutral promoters were ranked based on RNA expression levels of the associated genes. Promoters with the top 1000 or the bottom 1000 expressed genes were used as positives. ROC plots and area under the curve (AUC) values were calculated using the chromatin mark signals at promoter (log_2_(normalized counts + 1)) by the R plotROC (2.2.1).

### Enhancer epigenetic state analysis

To extract putative enhancer regions, the ATAC peaks of all cell types (macs2 -log10(q-value) >4) were merged by *bedtools merge* to generate a combined ATAC peak set. To pinpoint the summit of each combined peak, the ATAC summits of all cell types were first concatenated as one bed file and mapped to the combined ATAC peak set by *bedtools intersect*. For each combined peak that has more than one summit, the summit with the most significant macs2 q-value was chosen. Any combined ATAC peaks that overlapped promoters (TSS ± 1 kb) were removed and the distal ATAC peak summits were extended by ± 500 bp to generate the putative enhancer set. Any overlapping putative enhancers were merged by *bedtools merge*, resulting in a total of 150,464 putative enhancers.

To track the epigenetic state of enhancers, we defined enhancers as active, mixed, primed, poised, repressed and neutral based on the overlap with H3K4me1, H3K27ac and H3K27me3 peaks (at least 20% overlap by enhancer length) in each cell type as depicted in Fig. 2a. Enhancers without any H3K4me1, H3K27ac or H3K27me3 peaks were defined as ‘neutral’. Alluvial plots which track the epigenetic state transition of individual enhancer across cell types were generated using the R *ggalluvial* (0.12.3).

To study enhancer epigenetic dynamics (Fig. 2c), read counts of ATAC, H3K4me1, H3K4me3, H3K27ac and H3K27me3 at enhancer regions were extracted by featureCounts (options: ‘-f -p -O’) and normalized by featureCounts using relative read depth between libraries as size factors. To identify dynamically active enhancers, enhancers that were active in any cell types and exhibited differential H3K27ac signals (absolute[log2(signal fold change)] >1 and adjusted p-value <0.05) (Extended Data Fig. 2d) were extracted (constitutively active enhancers in all six cells types were excluded). This resulted in 21,652 dynamically active enhancers which were subjected to k-means clustering using the R *kmeans* function. The histone modification enrichment pattern at distal ATAC peak of each cell type was assessed by meta-accessible chromatin profile plot and heatmaps using deepTools *computeMatrix* and *plotHeatmap* with k-means clustering.

### Assignment of enhancers to genes

Each of the 150,464 enhancers were assigned to the nearest gene (distance to TSS <100 kb) using BETA(1.0.7)^96^. Since distance-based enhancer-gene assignment approach generates many false positive associations, we identified high-confidence enhancer-gene pairs using the strategy described by Gorkin et al. (2020) with modifications. Briefly, all of the enhancer-gene pairs were evaluated in terms of Kendall Rank Correlation coefficient (Kendall’s Tau) between the H3K27ac signals at enhancers and expression levels of the associated genes across the 12 sample sets (6 cell types and 2 replicates each). To calculate the p-values of each correlation, a null distribution was estimated empirically by calculating the Kendall’s Tau of the enhancer with all the genes on the chromosome. An empirical p-value was defined as the number of times an equal or better than the observed Kendall’s Tau was found in the null distribution. We identified a total of 11,620 high-confidence enhancer-gene pairs (p-value ≤ 0.05 and a Kendall’s Tau ≥ 0.3) which were used in gene ontology terms enrichment analysis.

### Transcription factor ChIP-seq data processing

Since ChIP-seq dataset of HA-SOX17 and myc-PRDM1 consisted of single-end and paired-end libraries, only read 1 of pair-end libraries was used for analysis. Raw single-end reads of different libraries were trimmed to 50 bp by Cutadapt. Subsequently, HA-SOX17, myc-PRDM1 (this study) and TFAP2C (GSE140021)^46^ reads were quality- and adaptor-trimmed by *Trim Galore*. The trimmed ChIP-seq and input reads were aligned to the human reference genome (UCSC hg38) by the *bwa aln* command of the Burrows–Wheeler Aligner(v0.7.17-r1188)^97^. Samtools *view* was used to remove unmapped and low-mapping quality reads (options: ‘view -F 4 -q 20’). Duplicated reads were removed by *samtools rmdup*. Reads mapped to non-canonical hg38 contigs and mitochondrial DNA (chrM) were removed by *samtools view*. Reads mapped to hg38 blacklisted regions were eliminated using *bedtools subtract*.

For peak calling and generation of bigwig signal tracks, ‘cleaned’ ChIP-seq and input libraries were subsampled using *samtools view* so that each library contains approximately the same number of reads. Peaks were called on the individual downsampled libraries and the merged downsampled libraries of the two replicates using macs2 *callpeak* against the corresponding inputs (options: ‘-g 3e9 – keep-dup all’). To evaluate the ChIP enrichment levels, the percentage of reads in peak was calculated using featureCounts. For visualization in IGV genome browser, the individual and merged downsampled libraries were converted into signal tracks using deepTools *bamCoverage* with reads per kilo base per million normalization (RPKM) normalization (options: ‘--binSize 10 --normalizeUsing RPKM --ignoreForNormalization chrX chrY –extendReads *’). The reads extension size (*) was calculated by macs2 in the peak calling step. For simplicity, the signal track and peak set of the merged replicates was used in subsequent analysis.

To cluster SOX17 and PRDM1 peaks (Fig. 3c), the two peak sets were combined by *bedtools merge*. Log_2_(ChIP/input) signal tracks were generated by WiggleTools(v1.2)^98^ and k-means clustering heatmaps at combined peaks were generated using deepTools *computeMatrix* and *plotHeatmap*. For peak distribution analysis, distance between the summit of TF peaks and the nearest TSS of protein coding and lincRNA genes (Gencode Human Release 30 basic gene annotation) was extracted using the *annotatePeaks.pl* script of HOMER.

Reads for OTX2 MNChIP-seq data^64^ (GSE61475) were aligned to human reference genome (GRCh38) using Bowtie2 using --local --very-sensitive options. Reads were deduplicated and replicates merged and normalised to counts per million (CPM) using deepTools bamCoverage using a bin size of 20. Peak calling was done using MACS2 using a q-value of 0.05.

### Identification of direct target genes of SOX17, PRDM1 and TFAP2C

To determine the direct targets of SOX17 and PRDM1 in gain-of-function experiments (Fig. 3), integrated TF ChIP-seq and transcriptome analysis was carried out using BETA. Briefly, SOX17/PRDM1 peaks were assigned to the nearby genes (distance to TSS from peak summit ≤ 100 kb) with the *BETA plus* command, which also infers direct target genes by integrating the differentially expressed genes in 12h PreME aggregates after SOX17/PRDM1 overexpression (absolute[log_2_(fold change)] >1 and adjusted p-value <0.05 between overexpression and control 12h PreME aggregates). A regulatory potential, which is a gene's likelihood of being regulated by a factor, is estimated for each gene^96^. The higher the regulatory potential, the shorter is the distance between the peak summit and the TSS of the associated genes. To predict the activating and repressing function, genes were divided into upregulated, downregulated and unchanged according to their expression patterns upon SOX17 or PRDM1 overexpression. Cumulative distribution function plot was generated for each group with genes ranked by decreasing regulatory potential. A one-tailed Kolmogorov-Smirnov test (R *ks.test* function) was used to determine the statistical significance between the differentially expressed groups and the unchanged group.

To determine SOX17, PRDM1 and TFAP2C cooperativity in hPGCLCs, peaks of the three TFs were merged to generate a combined peak set. Intersection of peaks and generation of venn diagram were performed using the R *Vennerable*(3.1.0.9000) (https://github.com/js229/Vennerable). The combined peaks were assigned to genes (distance to TSS from peak summit ≤ 100 kb) using *BETA minus*. Direct up target genes were defined as follows: 1) genes that were downregulated in *TFAP2C*/*SOX17*/*PRDM1* knockout hPGCLCs/aggregates (log_2_(fold change ) versus the wild-type control <1 and adjusted p-value <0.05) alone or cooperatively as indicated; 2) had the corresponding TFAP2C/SOX17/PRDM1 peak(s) within 100 kb of the TSS; and 3) the associated TF peak(s) overlapped with ‘active’ or ‘mixed’ enhancer or promoters in hPGCLCs. Similarly, direct down target genes were defined as 1) genes that were upregulated in *TFAP2C*/*SOX17*/*PRDM1* knockout hPGCLCs (log_2_(fold change) versus the wild-type control <1 and adjusted p-value <0.05) alone or cooperatively as indicated; 2) had the corresponding TFAP2C/SOX17/PRDM1 peak(s) within 100 kb of the TSS; and 3) the associated TF peak(s) did not overlap with ‘active’ enhancer or promoters in hPGCLCs.

### Gene ontology term, transcription regulator motif and binding site enrichment analysis

Gene ontology terms enrichment analysis was based on the Database for Annotation, Visualization and Integrated Discovery (DAVID) v6.8^81^ using the *RDAVIDWebService*(1.24.0). Motif enrichment analysis was performed using the HOMER *findMotifsGenome.pl* script. Motif search was restricted to DNA sequence ± 100 bp from ATAC/TF peak summits. Transcriptional regulators binding site enrichment analysis was based on the ReMap2020 database which contains DNA binding maps of 1,135 transcriptional regulators (TRs)^42^. Enrichment was calculated using the R *ReMapEnrich*(0.99.0) (https://github.com/remap-cisreg/ReMapEnrich). Promoter binding site enrichment analysis was carried out using all promoter regions (TSS ± 1 kb of protein-coding and lincRNA transcripts) as background.

### Luciferase reporter assay

Genomic regions containing enhancer (chr6:106,079,826-106,081,103) and promoter (chr6:106,085,395-106,086,553) of *PRDM1* were amplified from hESC genomic DNA. The wild-type enhancer and promoter were cloned into a PiggyBAC-based luciferase (Luc+) reporter plasmid containing a hygromycin resistant gene driven by a PGK promoter. Subsequently, the SOX motifs (ATTGT) in the enhancer (3x) and/or promoter (2x) were mutated into AGCAC by incorporating substitution mutations into PCR primer sequences circularised using the In-Fusion HD Cloning Plus kit (Takara). Using the Lipofectamine Stem Transfection Reagent (Invitrogen), each reporter plasmid was transfected into NANOS3–tdTomato reporter hESCs, together with a PiggyBAC plasmid containing a constitutively expressed renilla luciferase (Rluc) cassette and a neomycin resistant cassette, a PiggyBAC plasmid containing a Dex-inducible *SOX17* transgene and a puromycin resistant cassette^9^, and a plasmid encoding a PiggyBAC transposase. Stable cell lines were generated following triple selection by hygromycin, neomycin and puromycin. Following 24h of ± Dex treatment in Essential 8 medium, cells were collected and subjected to luciferase activity assay using the Dual-Glo Luciferase Assay System (Promega). Normalized luciferase activities were obtained by dividing firefly luciferase activity by renilla luciferase activity.

### CRISPR activation

We designed a CRISPRa plasmid and a single guide RNA (sgRNA) plasmid (Extended Data Fig. 6a) based on the dCas9-SunTag-VP64 system^54^. For the CRISPRa plasmid, we replaced the CMV promoter in the PB-CMV-MCS-EF1α-Puro PiggyBac cDNA Cloning and Expression Vector (SBI System Biosciences) by a TRE3G promoter (Takara). The dCas9-GCN4x5-P2A-scFV- superfolder green fluorescent protein (sfGFP) fragment from the pPlatTET-gRNA2 plasmid (Addgene, 82559) was amplified and inserted downstream of the TRE3G promoter. Finally, a synthetic VP64-GB1-NLS fragment (Integrated DNA Technologies) based on the pHRdSV40-scFv-GCN4-sfGFP-VP64-GB1-NLS vector (Addgene, 60904) was inserted downstream of the sfGFP. The resulting vector encodes a Dox-inducible SunTag system which consists of a catalytically inactive Cas9 (dCas9) fused to five GCN4 peptides separated by an optimized 22-amino-acid linkers^55^ and a scFV-sfGFP-VP64 transactivator fusion peptide which can be recruited to the dCas9 through the scFV-GCN4 domains. The system is completed with PiggyBAC gRNA plasmid which entails a sgRNA cassette driven by an U6 promoter and a Tet-On 3G-IRES2-Neomycin resistance cassette driven by an EF1α promoter. To improve sgRNA expression level and stability, we adopted an optimized scaffold sequence with an A-U basepair flip in the sgRNA stem-loop and an extended hairpin structure as described before^56^. 3-5 sgRNAs targeting the *SOX17*, *TFAP2C* and *PRDM1* enhancers, promoters and neutral regions, as well as 3 non-targeting sgRNA controls, were designed using the Custom Alt-RCRISPR-Cas9 guide RNA design tool of Integrated DNA Technologies (https://eu.idtdna.com/site/order/designtool/index/CRISPR_SEQUENCE) or selected from a previous publication^99^ (Supplementary Table 7).

The piggyBAC-based CRISPRa (puromycin resistant cassette) and sgRNA plasmids (neomycin resistant cassette), together with a plasmid encoding a hyperactive piggyBAC transposase, were co-transfected into a hESC line harbouring a NANOS3-tdTomato reporter using the Lonza 4D-Nucleofector transfection device. Stable cell lines with integration of the CRISPRa and sgRNA transgenes were generated after puromycin and neomycin selection for 7-10 days. To activate the enhancers and/or promoters, cells were treated with 0.5 μg/ ml doxycycline in Essential 8 medium for 2 days and fixed for immunofluorescence analysis. Alternatively, sfGFP-positive cells were collected by FACS and subjected to quantitative reverse transcription PCR analysis.

To induce hPGCLCs with CRISPRa, hESC lines harbouring the indicated sgRNA expression cassettes were differentiated into PreME and ME. Trypsinised hESCs, PreME cells and ME cells were cultured as floating aggregate for 4 days in hPGCLC induction medium supplemented with 0.5 μg/ ml doxycycline with or without BMP4. The day 4 EBs were subjected to immunofluorescence or FACS of NANOS3-tdTomato-positive cells for RT-qPCR analysis. In case expression of an analysed transcript was not detectable by RT-qPCR due to its low expression level (e.g., *SOX17* expression in control hESCs (Fig. 7b)), a Ct value of 40 (maximum cycle number) was assigned.

### CRISPR interference

For CRISPRi, we used the CRISPRa plasmid as the backbone and inserted a KRAB-dCas9-ecDHFR and a IRES-EGFP fragment ^57^ downstream of the TRE3G promoter using the In-Fusion HD Cloning Plus kit (Takara). The resulting plasmid encodes a KRAB-dCas9 transgene under the tight transcriptional control of a Dox-inducible promoter and a protein destabilisation degron DHFR. The addition of Dox and trimethoprim (TMP) allow robust mRNA and protein expression of KRAB-dCas9 CRISPRi machinery that can be tracked by EGFP expression.

To generate CRISPRi targeting lines, NANOS3-tdTomato reporter hESCs were co-nucleofected with the piggyBAC-based CRISPRi (puromycin resistant cassette) and sgRNA plasmids (neomycin resistant cassette) (Supplementary Table 7), as well as a hyperactive piggyBAC transposase plasmid using the Lonza 4D-Nucleofector. To assure the stable integration for both the CRISPRi construct and the sgRNA transgenes cells were selected for 7 to 10 days of combined puromycin and neomycin treatment after nucleofection.

To functionally test the role of the specific enhancers and neutral regions on PGCLC specification, CRISPRi lines were first induced into PreME and then cultured as floating aggregate for 4 days in hPGCLC induction medium with or without 0.5 μg/ml doxycycline and 10 μM TMP to induce CRISPR interference. The day 4 embryoid bodies were analysed by FACS. Cells were first gated by EGFP status followed by quantification of hPGCLC induction efficiency in each population (EGFP+ or EGFP-) using the NANOS3-tdTomato reporter and antibody staining for PDPN-PECy7 (BioLegend 337014, 5 ul/M) or PDPN-BV421 (BD Biosciences 566456, 5 ul/M). Induction efficiency in EGFP+ (CRISPRi+) cells was first normalised by that in EGFP- cells in the same line and relative normalised induction efficiency between CRISPRi lines was calculated in reference to the non-targeting control line.

To functionally test the role of OTX2 on hPGCLC competence, *OTX2* promoter-targeting and non-targeting CRISPRi lines were pre-treated for 24h in E8 media followed by PreME induction with or without 0.5 μg/ml doxycycline and 10 μM TMP to induce CRISPR interference. PreME cells were trypsinised and cultured as floating aggregates for 4 days in hPGCLC induction medium without TMP and doxycycline. At day 4, embryoid bodies were analysed by FACS as described above.

### Generation of single-cell RNA-seq libraries

hESCs, PreME and ME cells were FACS sorted into PBS with 0.04% weight/volume BSA (400 μg/mL). Sorted populations were loaded into the 10x-Genomics Chromium using the single cell 3’ reagents kit v2. Libraries were prepared as per the manufacturer’s instructions and pooled for sequencing. Libraries were sequenced on an Illumina HiSeq 4000 (paired-end; read 1: 26 cycles; i7 index: 8 cycles, i5 index: 0 cycles; read 2: 98 cycles) aiming at a minimum coverage of 50,000 raw reads per cell.

### Single cell data processing and analysis

Multiplexed single-cell libraries were processed using the 10X Genomics cell ranger pipeline. Reads were aligned to a reference genome (Homo sapiens GrCh38) using STAR, and quantification of genes against an annotation reference (based on Ensembl GrCh38 v90). Initial analysis of our data was done using Seurat (v3.1.4). Count data was normalised and scaled using NormalizeData based on log counts per 10000 (logCP10k) and scaled using ScaleData. UMAP plots were calculated using the first 20 PCs. Diffusion maps were generated using destiny (2.12.0).

### Immunofluorescence

Adherent cells were cultured on ibidi μ-Slide and fixed in 4% PFA for 30 minutes at 4°C. Embryoid bodies were fixed in 4% PFA for 2h at 4°C and embedded in OCT compound for frozen sections. The samples were incubated with primary antibodies for overnight at 4°C and subsequently with fluorescence-conjugated secondary antibodies (Thermo Fisher Scientific) and DAPI for 1h at RT. The primary antibodies used were: anti-GFP (abcam ab13970, 1:1000), anti-PRDM1 (Cell Signaling Technology 9115, 1:200), anti-SOX17 (R&D AF1924, 1:500), anti-TFAP2C (Santa Cruz Biotechnology sc-8977, 1:200), and anti-OCT4 (BD Biosciences 611203, 1:500). Samples were imaged under Leica SP8 upright or inverted scanning confocal microscope and analysed using Volocity (6.3).

### Quantitative reverse transcription PCR

Total RNA was extracted using PicoPure RNA Isolation Kit (Thermo Fisher Scientific) and cDNA was synthesized using QuantiTect Reverse Transcription Kit (QIAGEN) according to manufacturer’s protocols. qPCR was performed on a QuantStudio 6 Flex Real-Time PCR Systems (Applied Biosystems) using SYBR Green JumpStart Taq ReadyMix (Sigma) and specific primers (Supplementary Table 7). The ΔΔCt method was used for quantification of gene expression.

### Western blot analysis

Western Blot analysis was performed as described before^100^. In brief, proteins were separated on a 10% polyacrylamide gel using the Mini-PROTEAN system (Bio-Rad) and transferred to an Immobilon-P transfer membrane (Millipore). After blocking in 5% skimmed milk, the membrane was cut according to the molecular weight marker and decorated with rabbit anti-H3 (Abcam ab1791, 1:10,000) and goat anti-OTX2 (R&D Systems AF1979, 1:1,000). Histone antibody binding was visualized using IRDye 680RD (LI-COR, 1:2,000) and the LI-COR Odyssey CLx system. OTX2 antibody binding was detected by horseradish peroxidase-conjugated anti-goat IgG (Dako; 1: 2,000 in 5% skimmed milk, 0.01% TBST) in conjunction with the Western Detection System (GE Healthcare).

### Statistics & reproducibility

For ChIP-seq, ATAC-seq and RNA-seq, two independent biological replicates were included according to guidelines of the Encode Consortium^101^. No statistical method was used to predetermine sample size in other experiments. Low quality replicate of ATAC-seq and ChIP-seq libraries were excluded from the analysis, as determined by percentage of reads in peaks, number of peaks, and genome browser visualisation. All results involved equipment-based quantitative measure and no subjective rating of data was involved, hence blinding is not relevant. Wilcoxon rank sum test was performed using R *ggpubr* (0.4.0). Hypergeometric test was performed using the R *phyper* command.

## 1 Extended Data

## Supplementary Material

Source data Figure 3

Source data Figure 5

Source data Figure 6

Source data Figure 7

Source data Figure 8

Source_data_Extended_data_figure 6

Source_data_Extended_data_figure 8

Supplementary tables 1-7

## Data Availability

Any enquiries on reagents and cell lines can be directed to (a.surani@gurdon.cam.ac.uk). CRISPRa/i plasmids are deposited to Addgene (183409, 183410 and 183411). Other plasmids generated in this study will be made freely available upon request. Modified human embryonic stem cell lines generated in this study will be made available on request upon completion of a Materials Transfer Agreement. ChIP-seq and RNA-seq datasets are available on NCBI GEO (GSE159654). Single cell sequencing datasets are available on ArrayExpress (E-MTAB-11135). Previously published data that were re-analysed here are: hPGC RNA-seq (GSE60138), TF knockout RNA-seq (GSE99350), TFAP2C ChIP-seq (GSE140021) and OTX2 ChIP-seq (GSE61475). Genome databases used are: UCSC GRCh38/hg38, Ensembl GrCh38 v90 and Gencode Human Release 30. Source data are provided with this study. All other data supporting the findings of this study are available from the corresponding author on reasonable request.
