# Supplementary material for: Sequential enhancer state remodelling defines human germline competence and specification
Source: Nat Cell Biol. Author manuscript; Available in PMC 2022 May 16. (PMC7612729; doi:10.1038/s41556-022-00878-z)
Supplement: Source_data_Extended_data_figure 8 [file EMS143940-supplement-Source_data_Extended_data_figure_8.pdf]

Western Blot: Extended Data Fig. 8c

12-05-2021 goat-anti-OTX2

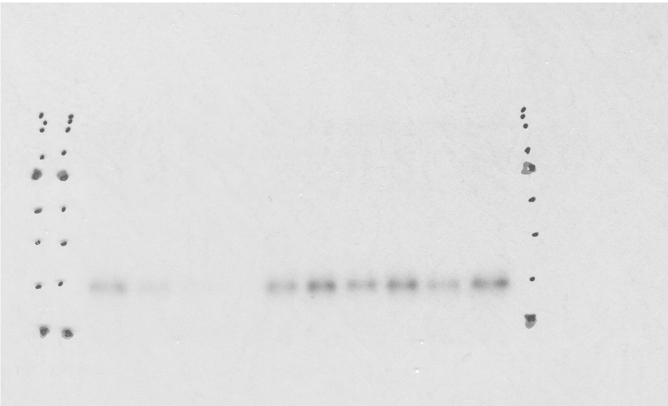

72 kD
55 kD
43 kD
34 kD
26 kD

M M 1 2 3 4 5 6 M

12-05-2021 rabbit-anti-H3

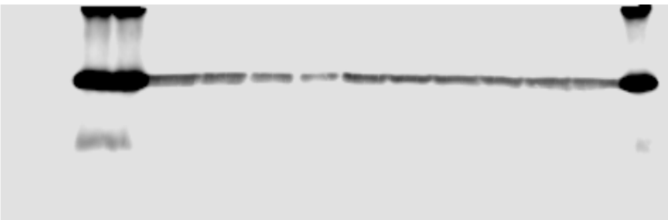

26 kD
17 kD

M M 1 2 3 4 5 6 M

M = marker
1 = 96 h + Doxycycline / TMP
2 = 96 h vehicle
3 = 72 h + Doxycycline / TMP
4 = 72 h vehicle
5 = 24 h + Doxycycline / TMP
6 = 24 h vehicle
